# BMI1 Silencing Induces Mitochondrial Dysfunction in Lung Epithelial Cells Exposed to Hyperoxia

**DOI:** 10.3389/fphys.2022.814510

**Published:** 2022-03-30

**Authors:** Helena Hernández-Cuervo, Ramani Soundararajan, Sahebgowda Sidramagowda Patil, Mason Breitzig, Matthew Alleyn, Lakshmi Galam, Richard Lockey, Vladimir N. Uversky, Narasaiah Kolliputi

**Affiliations:** ^1^Division of Allergy and Immunology, Department of Internal Medicine, Morsani College of Medicine, University of South Florida, Tampa, FL, United States; ^2^Department of Molecular Medicine, Morsani College of Medicine, University of South Florida, Tampa, FL, United States; ^3^Division of Epidemiology, Department of Public Health Sciences, College of Medicine, Pennsylvania State University, Hershey, PA, United States

**Keywords:** mitochondria, BMI1, acute lung injury (ALI), Acute Respiratory Distress Syndrome (ARDS), human lung epithelial cell

## Abstract

Acute Lung Injury (ALI), characterized by bilateral pulmonary infiltrates that restrict gas exchange, leads to respiratory failure. It is caused by an innate immune response with white blood cell infiltration of the lungs, release of cytokines, an increase in reactive oxygen species (ROS), oxidative stress, and changes in mitochondrial function. Mitochondrial alterations, changes in respiration, ATP production and the unbalancing fusion and fission processes are key events in ALI pathogenesis and increase mitophagy. Research indicates that BMI1 (B cell-specific Moloney murine leukemia virus integration site 1), a protein of the Polycomb repressive complex 1, is a cell cycle and survival regulator that plays a role in mitochondrial function. BMI1-silenced cultured lung epithelial cells were exposed to hyperoxia to determine the role of BMI1 in mitochondrial metabolism. Its expression significantly decreases in human lung epithelial cells (H441) following hyperoxic insult, as determined by western blot, Qrt-PCR, and functional analysis. This decrease correlates with an increase in mitophagy proteins, PINK1, Parkin, and DJ1; an increase in the expression of tumor suppressor PTEN; changes in the expression of mitochondrial biomarkers; and decreases in the oxygen consumption rate (OCR) and tricarboxylic acid enzyme activity. Our bioinformatics analysis suggested that the BMI1 multifunctionality is determined by its high level of intrinsic disorder that defines the ability of this protein to bind to numerous cellular partners. These results demonstrate a close relationship between BMI1 expression and mitochondrial health in hyperoxia-induced acute lung injury (HALI) and indicate that BMI1 is a potential therapeutic target to treat ALI and Acute Respiratory Distress Syndrome.

## Introduction

Acute Respiratory Distress Syndrome (ARDS) is characterized by the rapid onset of respiratory distress (usually unresponsive to therapy), a decrease of lung compliance and volumes, and loss of ability to regulate gas exchange and can trigger severe respiratory failure (Ferguson et al., 2012; Ranieri et al., 2012; Henderson et al., 2017). Acute Lung Injury (ALI), according to the Berlin definition, is a less serious category of ARDS with hypoxemia of 300 mm Hg ≥ PaO_2_/FiO_2_ > 200 mm Hg (Johnson and Matthay, 2010; Ranieri et al., 2012; Umbrello et al., 2016). ALI is one of the major causes of morbidity in individuals over 60 years of age with a mortality rate of ≥ 60% (Blank and Napolitano, 2011). It worsens with time and has comorbidities such as hydrodynamic imbalance, cor pulmonale, lung infections, atelectasis, pneumothorax, pulmonary embolism, and damage of other organs (Chapalamadugu et al., 2015; Ochiai, 2015; Sipmann et al., 2018). It affects 255,000 new subjects per year in the US and costs the healthcare system more than 8 billion dollars (Ochiai, 2015; Ruhl et al., 2015; Marti et al., 2016; Rezoagli et al., 2017).

Lung epithelial cells play a critical role in ALI. Type I alveolar epithelial cells (AECI) are responsible for gas exchange and type II alveolar epithelial cells (AECII) produce lung surfactant and antimicrobial factors. AECII are the most abundant cells in the alveoli (60–80%) and comprise 10–15% of total lung cells they are vital for respiratory function, contain 50% of the mitochondria within the lung, and are necessary for appropriate lung function (Massaro et al., 1975; Crapo et al., 1978, 1980). AECII are responsible for the epithelial renewal and the production of cytokines and chemokines following lung injury. As ALI progresses, the integrity of the alveolar-capillary barrier is compromised, gas exchange and the assimilation of oxygen within alveoli decrease, and the demand for supplemental oxygen increases. Oxygen toxicity becomes evident with the appearance of symptoms and signs after 24 h of oxygen therapy with an FiO_2 ≥_ 0.75, the lung damage results in hyperoxia-induced acute lung injury (HALI) (Cochrane et al., 1983; Kolliputi and Waxman, 2009a,b; Waxman and Kolliputi, 2009; Beasley, 2010; Kolliputi et al., 2010, 2012; Kallet and Matthay, 2013).

ALI is characterized by the activation of an innate immune response, an influx of macrophages and white blood cells, and consequently, the release of cytokines and chemokines into alveolar spaces (Goodman et al., 2003; Chuquimia et al., 2013). This is followed by alterations in mitochondrial function that increase reactive oxygen species (ROS), promote oxidative stress, induce alterations in the electron transport chain (ETC), and in turn cause more mitochondrial dysfunction (Goodman et al., 2003; Imai et al., 2008; Islam et al., 2012; Prockop, 2012). These events lead to epithelial cell death, thereby increasing lung permeability. Ultimately, this culminates in pulmonary edema and eventually, ALI. Both epithelial cell integrity and mitochondrial function are crucial in normal lung physiology.

Maintaining mitochondrial health and its bioenergetic balance is necessary in ALI treatment for which different studies have been carried out in animals, mainly in the mouse model of Lipopolysaccharide-induced Acute Lung Injury (LALI) (Islam et al., 2012; Shi et al., 2019; Zhang et al., 2020), today research points toward translational studies using mitochondrial therapy to improve human health (McCully et al., 2016, 2017; Yamada et al., 2020). New therapy is targeting inherited or acquired mitochondrial diseases and other pathological conditions where mitochondrial metabolism and biogenesis are compromised or there is increased mitophagy (Wolf et al., 2015; Gorman et al., 2016; Hirano et al., 2018; Viscomi and Zeviani, 2020; Weissig, 2020; Willmes, 2020; Weissig and Edeas, 2021). A few proposed treatments include: the use of vitamins and cofactors, antioxidants, mitochondrial gene therapy, mitochondrial transplantation (replacement therapy), regulation of mitochondrial dynamics, bypassing of the mitochondrial genome, activation of mitochondrial biogenesis, and regulation of mitophagy (Wolf et al., 2015; McCully et al., 2016, 2017; Garone and Viscomi, 2018; Hirano et al., 2018; Weissig and Edeas, 2021).

Mitochondrial research uses different biomarkers, from proteins belonging to structure and metabolism, to external regulators and recruiters that condition mitochondrial function (Prockop, 2012; Yamada et al., 2020). Polycomb complex protein BMI1 (B cell-specific Moloney murine leukemia virus integration site 1), a ubiquitous protein widely studied for producing chromatin acetylation and being overexpressed in some types of cancer, is also involved in mitochondrial metabolism. In global BMI1 knockout mice, decreased expression is associated with an increased production of mitochondrial ROS, alterations in the ETC, and activation of the DNA Damage Response pathway (DDR) (van der Lugt et al., 1994; Baughman and Mootha, 2006; Liu et al., 2009; Bhattacharya et al., 2015). The mechanism by which this occurs is not yet elucidated. BMI1 is necessary for lung stem cell renewal and repair of lung tissue following lung injury and oxidative stress; however, the downstream effects of abnormally low BMI1 expression in lung tissue remain unknown. In addition, BMI1 is involved in autophagy-mediated necroptosis and the maintenance of hematopoietic function (Park et al., 2003; Dey et al., 2016). The former is a type of programmed cell death related to mitophagy and involves changes in the expression of PINK1 (PTEN-induced kinase 1), Parkin, and DJ1 (Protein deglycase DJ1), all closely linked to the PI3K/Akt-mTOR signaling pathway and to alterations in TCA enzyme activity (Ryter and Choi, 2010; Aggarwal et al., 2016; Shi et al., 2019; Zhang et al., 2020). BMI1 expression decreases under hyperoxic conditions. Based on this, and the aforementioned involvement of BMI1 in DDR and the PI3K/Akt-mTOR signaling pathway, we posit that BMI1 could be a mediator of the organ damage associated with HALI, akin to the mechanisms previously demonstrated in cardiomyocytes and stem cells (Dong et al., 2014; Valiente-Alandi et al., 2015, 2016; Lee et al., 2016; Herrero et al., 2018a,b).

This study focuses on utilizing a hyperoxia model to mimic ALI in H441 cells (human epithelial lung cells) to assess the mechanistic role of BMI1 in mitochondrial function as it relates to oxidative stress, reactive oxygen species production, and alterations in the oxygen consumption rate (OCR).

## Results

### Hyperoxia Induces a Decrease in BMI1 Levels in Epithelial Lung Cells

**FIGURE 1:**
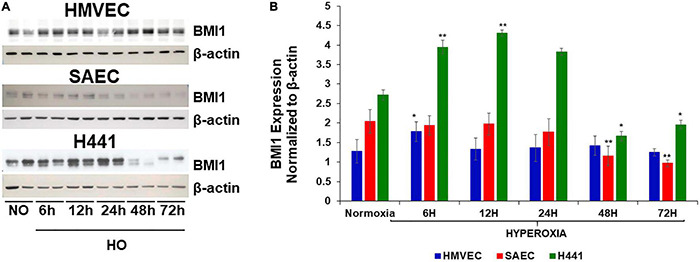
BMI1 expression in three cell lines was evaluated at different time points of hyperoxia exposure. **(A)** Western blot of BMI1 expression after different time points of hyperoxia exposure. **(B)** Densitometric analysis of BMI1 expression after normalization to β-actin. *n* = 6 (mean ± SEM), one-way ANOVA, *p ≤ 0.05, **p ≤ 0.01.

Small Airway Epithelial Cells (SAECs), H441 lung epithelial cells, and Human Lung Microvascular Endothelial Cells (HMVEC-L) were exposed to normoxic (NO) vs. hyperoxic (HO) conditions to determine BMI1 expression. Western blot analysis in whole cell lysate indicates that the levels of BMI1 protein decreased significantly in epithelial SAEC and H441 cells after 48 h of HO (Figures 1A,B). There were no significant changes observed in endothelial cells (Figure 1A) indicating that BMI1 expression is affected by high oxygen concentration in lung epithelial but not endothelial cells. The three cell types were exposed to NO or HO at different time points (6–72 h) to determine if the expression of BMI1 is related to ALI. BMI1 levels increased in H441 cells during the first 12 h of hyperoxia exposure (Figure 1B). Cox et al. (2015a) showed that the increased antioxidant protein expression at 12 h of HO is correlated with apoptosis resistance, cell proliferation and growth, and inhibition of ROS accumulation as a possible first response to the injury in epithelial cells. No changes in the expression of BMI1 were evident after 24 h of HO vs. NO exposure. The expression of BMI1 in SAECs is relatively unchanged in the first 24 h and decreases progressively during the oxygen exposure period. Interestingly, BMI1 expression decreased significantly in SAECs and H441 cells after 48 h of hyperoxia, when oxidative stress measured by an increase in ROS production and autophagy occur (Figures 1A,B; Cox et al., 2015a,b). Based on these findings, the authors question whether BMI1 is necessary for a response to oxidative stress in hyperoxia model of ALI. Therefore, a pool of anti-BMI1 siRNAs was used, and the best sequence was chosen to maximize the knockdown of BMI1 expression in H441 cells.

### BMI1 Is Necessary to Respond to Hyperoxia in *in vitro*

#### Mitochondrial Dynamics Markers

**FIGURE 2:**
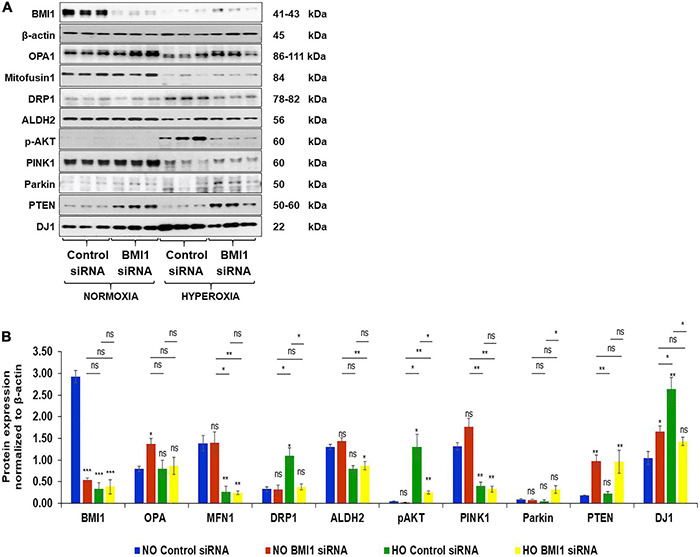
Expression levels of proteins related to mitochondrial dynamics and metabolism in Control and BMI1 silenced H441 Cells. **(A)** Western blot analysis of proteins in normoxia (NO) and hyperoxia (HO). **(B)** Densitometric analysis of protein expression after β-actin normalization. *n* = 6, mean ± SEM; ns = no significant, *p ≤ 0.05, **p ≤ 0.01, ***p ≤ 0.001.

The expression of mitochondrial proteins (fusion, fission, etc.) was evaluated in NO and HO conditions (Figures 2A,B) to evaluate the impact of silencing BMI1 expression in mitochondria metabolism. The results show that the expression of OPA1 (mitochondrial dynamin like GTPase) increases in BMI1 silenced cells under both NO and HO conditions, with significant difference in expression under NO (*p* = 0.040) (Table 1). Mitofusin 1 and 2 expression decreases after exposure to HO, but their expression did not change significantly after BMI1 silencing. This indicates that mitofusin 1 and 2 expression is modified by the oxygen concentration but not by BMI1 silencing (Figure 2B). This study also demonstrates significant changes in DRP1 (dynamin like-1 protein) expression with hyperoxia. DRP1 is a fission protein that facilitates mitophagy (Han et al., 2020). The expression of DRP1 is influenced by the oxygen concentration (NO vs. HO) (Figures 2A,B). An additive effect was observed between BMI1 silencing and an increase in oxygen concentration, *p* = 0.016 for O_2_*siRNA (Figures 2A,B and Table 1), evidenced by the decrease in the expression of DRP1 when BMI1 was silenced in H441 cells and exposed to HO. Aldehyde dehydrogenase 2 (ALDH2), a mitochondrial protein involved in the oxidative metabolism of alcohol and a potential shield against oxidative stress (Sidramagowda Patil et al., 2021), showed no significant changes following BMI1 silencing.

**TABLE 1:** Summary of *P*-values of two-way MANOVA for protein expression levels.

Independent variable	Dependent variable								
	OPA	MFN1	DRP1	ALDH2	pAKT	Pink1	Parkin	PTEN	DJ1
O_2_ Concentration	0.089	0.000	0.007	0.032	0.001	0.000	0.061	0.934	0.004
siRNA	0.040	0.977	0.015	0.835	0.006	0.146	0.028	0.001	0.119
O_2_ and siRNA	0.147	0.932	0.016	0.315	0.008	0.050	0.016	0.884	0.001

#### Regulatory Proteins of Mitochondrial Function

Proteins involved in the PI3K/AKT pathway as well as mitochondrial regulator proteins (widely studied in aging-associated diseases such as Parkinson’s disease and Alzheimer’s disease) were evaluated after BMI1 silencing to further elucidate the mechanism by which BMI1 acts in mitochondrial function. The expression of pAKT (Ser473), Pink1, Parkin, and DJ1 was regulated for both oxygen concentration and silencing of BMI1 (Table 1 and Figures 2A,B). The corresponding increase in pAKT and Parkin levels was driven by BMI1 silencing while changes in the expression of Pink1 and DJ1 were regulated by the oxygen concentration (Figures 2A,B). PTEN, a tumor suppressor that inhibits the PI3K/AKT survival pathway, had a higher level of expression when BMI1 was silenced, irrespective of hyperoxia exposure, evidencing the pivotal role of BMI1 in cell survival and proliferation (Figures 2A,B and Table 1).

These findings show that the silencing of BMI1 increases the expression of PTEN, Pink1, and DJ1, under NO conditions, resulting in mitophagy. PTEN expression remained elevated after HO in silenced cells, Parkin expression increases and Pink1 and pAKT decrease. The combination of these protein changes contributes to the inhibition of the PI3K-AKT pathway and the activation of mitophagy (Table 2). BMI1 expression regulates the protein levels of Pink1-Parkin-DJ1 (proteins involved in mitochondrial quality control, mitochondrial ubiquitination and mitophagy). Our study shows that BMI1 protein levels decrease significantly after hyperoxia exposure in H441 cells. In addition, BMI1 silencing in H441 cells induces mitochondrial dysfunction under normoxia and hyperoxia. Based on our data (Table 2 and Figure 2), we conclude that knockdown of BMI1 in H441 cells results in a loss of mitochondrial membrane potential (ΔΨm), increases mitophagy and cell death (Table 2 and Figure 2).

**TABLE 2:** Effect of Hyperoxia and BMI1 silencing in protein expression levels of mitochondrial biomarkers and regulatory proteins of mitochondrial function.

Protein	Treatment		
	BMI1 silencing	Hyperoxia	BMI1 silencing and Hyperoxia
Mitofusin 1	≈	↓	↓
OPA1	↑↑	↓	↑
DRP1	≈	↑	↑
ALDH2	≈	↓	↓
Pink1	↑	↓↓	↓
Parkin	↓	≈	↑↑
DJ1	↑	↑	↑
PTEN	↑	≈	↑
pAKT	↓	↑↑	↑

### BMI1 Silencing Changes the Levels of Gene Transcripts in a Different Manner Relative to the Levels of Protein Expression

**FIGURE 3:**
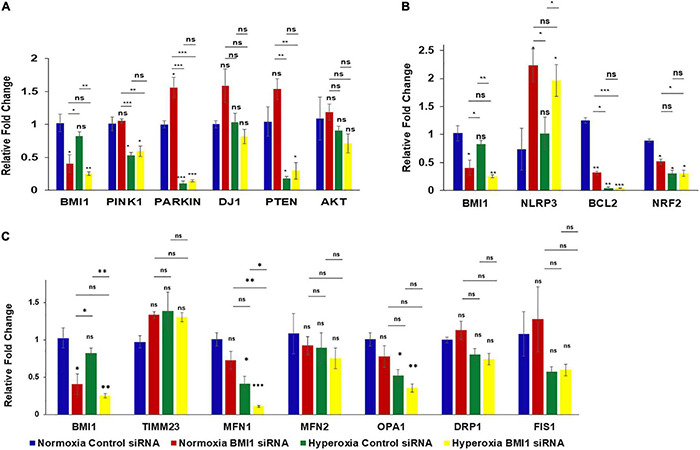
Analysis of transcript levels in BMI1 silenced H441 cells exposed to hyperoxia. **(A)** qRT-PCR analysis of transcripts involved in mitophagy pathway in BMI1 silenced vs. non-silenced H441 cells exposed to NO and HO. **(B)** qRT-PCR analysis of transcripts related to ALI in BMI1 silenced vs. non-silenced H441 cells exposed to NO and HO. **(C)** qRT-PCR analysis of transcripts related to mitochondrial dynamics in BMI1 silenced vs. non-silenced H441 cells exposed to NO and HO. All the four groups were compared to each other using MANOVA. Data represented as mean ± SEM, *n* = 3, ns = not significant, *p ≤ 0.05, **p ≤ 0.01, ***p ≤ 0.001.

Gene transcripts were analyzed through quantitative RT-PCR, using TaqMan RT-qPCR probes and β-actin as the internal calibrator to better understand if the impact of BMI1 silencing on protein expression was regulated at the transcriptomic level. Data, analyzed by two-way multivariate analysis of variance (MANOVA) (Table 3), show that oxygen concentration significantly decreased (*p* < 0.05) the expression levels of gene transcripts encoding proteins involved in mitophagy (Pink1, Parkin, and DJ1) and cell survival (PTEN, BCL2, and NRF2). Moreover, a combination of high O_2_ concentration and BMI1 silencing diminish the transcript levels of TIMM23, Parkin, DJ1 and BCL2. However, simple comparisons analysis (Figure 3 and Table 4) shows that silencing of BMI1, in combination with hyperoxia, induces a significant decrease in transcripts of *TIMM23*, genes encoding for mitochondrial fusion proteins (MFN1 and OPA1), for proteins involved in mitophagy (Pink1, Parkin), in cell survival (PTEN), anti-apoptosis (BCL2), and oxidation (NRF2), as well as increases the transcript levels of *NLRP3* gene encoding the NLRP3 inflammasome protein. Nevertheless, the transcript levels did not correspond with their protein expression levels, indicating that posttranscriptional modifications and other pathway signals are implicated in the regulation of protein synthesis.

**TABLE 3:** Summary of *P*-values of two-way MANOVA for transcript levels.

Independent variable	Dependent variable														
	BMI1	TIMM23	MFN1	MFN2	OPA1	DRP1	FIS1	Pink1	Parkin	DJ1	PTEN	AKT	NLRP3	BCL2	NRF2
O_2_ Concentration	0.071	0.003	0.000	0.374	0.002	0.319	0.058	0.000	0.000	0.046	0.000	0.122	0.987	0.000	0.001
siRNA	0.000	0.850	0.011	0.464	0.082	0.660	0.669	0.477	0.007	0.275	0.080	0.806	0.001	0.000	0.115
O_2_ and siRNA	0.813	0.036	0.893	0.962	0.759	0.920	0.754	0.895	0.016	0.034	0.249	0.464	0.255	0.000	0.263

**TABLE 4:** Effect of Hyperoxia and BMI1 silencing in gene transcripts levels based on simple comparisons analysis.

Transcript	Treatment		
	BMI1 silencing	Hyperoxia	BMI1 silencing and Hyperoxia
BMI1	↓	≈	↓↓
TIMM23	↑	≈	↓↓
Mitofusin 1	≈	↓	↓↓↓
Mitofusin 2	≈	≈	≈
OPA1	≈	↓	↓↓
DRP1	≈	≈	≈
FIS1	≈	≈	≈
Pink1	≈	↓	↓
Parkin	↑	↓↓↓	↓↓↓
DJ1	≈	≈	≈
PTEN	≈	↓	↓
AKT	≈	≈	≈
NLRP3	↑	≈	↑
BCL2	↓↓	↓↓	↓↓↓
NRF2	↓	↓	↓

### BMI1 Silencing Interferes With Mitochondrial Respiration

**FIGURE 4:**
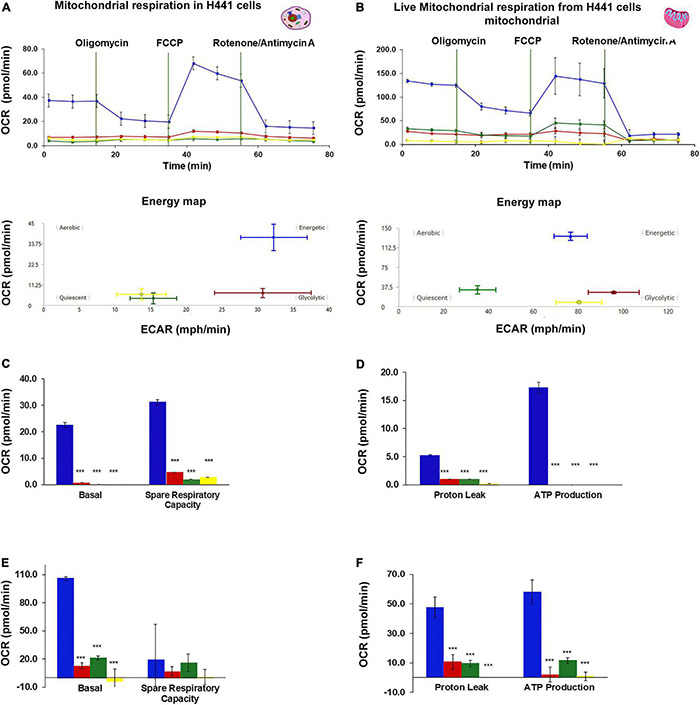
Oxygen Consumption Rate (OCR) in H441 cells and live mitochondria-derived from H441 cells. **(A,C,E)** OCR in intact H441 cells, **(A)** mitochondrial respiration and energy map, **(C)** basal respiration and spare respiratory capacity, **(E)** proton leak and ATP production. H441 cells were seeded at a density of 5 × 10^4^ cells per well of 96 wells Seahorse Agilent plate, 20 wells were analyzed for each treatment, graphs represent mean ± SEM, one-way ANOVA, ***p ≤ 0.001. **(B,D,F)** OCR in live mitochondria-derived from H441 cells, **(B)** mitochondrial respiration and energy map, **(D)** basal respiration and spare respiratory capacity, **(F)** proton leak and ATP production. A total of 1,000,000 particles (mitochondria) were seed in each well of 96 Seahorse Agilent plate, 8 wells were analyzed for treatment, graphs represent mean ± SEM, one-way ANOVA, ***p ≤ 0.001.

An analysis of OCR was performed on H441 cells and their live mitochondria to determine if BMI1 silencing affects mitochondrial function. In both cases, the consumption of oxygen was significantly higher in non-silenced cells under normoxic conditions than in BMI1 silenced cells exposed to hyperoxia (Figures 4A,B; ^***^p < 0.001). The basal respiration level was around 20.0 pmol/min for non-silenced cells in NO but was almost undetectable in cells exposed to HO and/or BMI1 silencing (Figures 4A,C). The energy map indicated that these cells had an energetic phenotype (Figure 4A), producing approximately 17 pmol/min of ATP as their main energy source (Figure 4E). Silenced H441cells in NO utilized glycolysis as an energy source while the cells exposed to HO turned to the quiescent phenotype (Figure 4A- energy map).

Live mitochondria derived from the non-silenced cells in NO had a significantly higher consumption of oxygen (110 pmol/min) than mitochondria derived from silenced cells or from cells exposed to hyperoxia (Figures 4B,D). Mitochondria from non-silenced cells in NO showed an energetic phenotype with higher ATP production (60 pmol/min) (Figure 4F). An OCR vs. time curve for the live mitochondria showed a similar trend to that observed in the live cells. The absolute values of basal respiration, spare respiratory capacity, ATP production and proton leak were threefold higher for live mitochondria isolated from non-silenced cells in NO and HO than for live H441 cells under the same oxygen conditions (Figures 4A,C,E). However, the consumption of O_2_ by cells and live mitochondria obtained from silenced cells in hyperoxia did not show differences in magnitude, and the energy map for mitochondria derived from H441 cells silenced for BMI1 and exposed to hyperoxia demonstrated a quiescent phenotype in terms of oxygen consumption (same that was shown for H441 cells) (Figure 4).

### BMI1 Silencing and Hyperoxia Promote Mitochondrial Dysfunction in the Enzymes From the Tricarboxylic Acid Cycle

**FIGURE 5:**
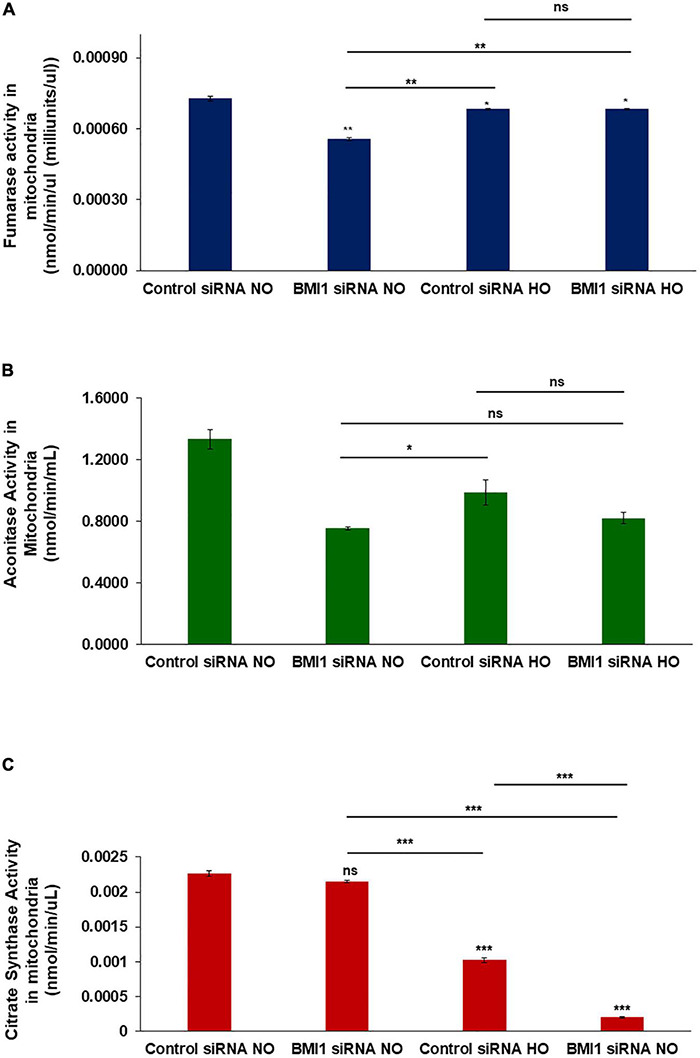
Mitochondria enzymatic activity in BMI1 silenced H441 cells exposed to hyperoxia. **(A)** Fumarase activity (nmole/min/μL) in mitochondrial lysates from BMI1 silenced or non-silenced H441 cells exposed to normoxia and hyperoxia. **(B)** Aconitase activity (nmole/min/mL) in mitochondrial lysates from BMI1 silenced or non-silenced H441 cells exposed to normoxia and hyperoxia. **(C)** Citrate synthase (nmole/min/μL) in mitochondrial lysates from BMI1 silenced or non-silenced H441 cells exposed to normoxia and hyperoxia. *n* = 3, mean ± SEM; two-ways MANOVA ns = non-significant *p ≤ 0.05, **p ≤ 0.01, ***p ≤ 0.001.

Mitochondria from H441 cells were isolated after treatment with control or BMI1 siRNAs, followed by NO vs. HO treatment to investigate mitochondrial functionality. Three different activity assays were performed: 1. fumarase activity in mitochondria decreased significantly, both after BMI1 silencing (*p* = 0.0014) and under HO conditions (*p* = 0.024) (Figure 5A); 2. aconitase activity decreased significantly in mitochondria, providing evidence for additive deleterious effects when BMI1-silenced cells are expose to hyperoxia (Figure 5B). The decrease in aconitase activity, in both NO and HO, was significantly greater after BMI1 silencing vs. non-silencing; and 3. citrate synthase activity did not show differences between mitochondria isolated from H441 cells exposed to normoxic conditions (silenced for BMI1 or non-silenced). However, compared to normoxia, the activity decreased substantially in mitochondria derived from H441 cells exposed to hyperoxia, and this reduction was worse in mitochondria from cells subjected to both hyperoxia and BMI1 silencing (Figure 5C). The results of these enzymatic activity assays demonstrate that BMI1 is necessary for mitochondria to respond to the damage caused by hyperoxia and to properly carry out the TCA cycle (tricarboxylic acid cycle/Krebs cycle).

### Oxidative Stress and Reactive Oxygen Species Production Increases After BMI1 Silencing, Leading to Mitochondrial Dysfunction

**FIGURE 6:**
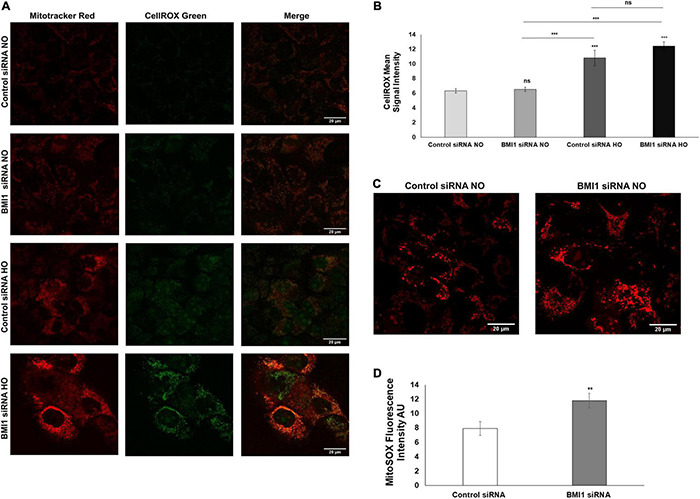
Analysis of mitochondria oxidative stress in BMI1 silenced H441 cells. **(A)** BMI1 silenced and non-silenced h441 cells were stained with CellROX green and mitochondria were colocalized with MitoTracker Red. **(B)** Images were quantified and represented as CellROX mean signal intensity. **(C)** Reactive oxygen species (ROS) were detected in BMI1 silenced and non-silenced h441 in normoxia using MitoSOX Red (superoxide ion). A representative image is shown. **(D)** Quantification of ROS production in BMI1 silenced and non-silenced cells in normoxia is represented as Mito SOX = x fluorescence intensity (AU). Data represents mean ± SEM; two-ways MANOVA ns = no significant **p ≤ 0.01, ***p ≤ 0.001.

Contrast confocal microscopy image analysis was used to determine the mitochondrial phenotype and function to characterize the mitochondria of H441 cells after BMI1 silencing. MitoTracker™ red and Cell-Rox™ green dyes were used to detect mitochondria and oxidative stress, respectively, thus allowing for co-localization. H441 cells were more readily detached after BMI1 silencing and hyperoxia, and oxidative stress was significantly higher after exposure to hyperoxia vs. normoxic conditions (as evidenced by the increase in green fluorescence) (Figures 6A,B). Superoxide ion production was assessed using MitoSox™ red dye to look for detectable changes in mitochondrial function. This analysis reveals that cells in which BMI1 is silenced produce significantly more ROS, as evidenced by an increase in red fluorescence (Figures 6C,D).

### Computational Analysis of the Prevalence and Functionality of Intrinsic Disorder in BMI1 Protein

#### Structural Features of BMI1

Observations done in this study and previous ones define BMI1 as a multifunctional protein suggesting that it belongs to the group of intrinsically disordered proteins (Wright and Dyson, 1999; Dunker et al., 2001, 2002a,2002b,^2005^). Data presented in Supplementary Material and Supplementary Figure 1, shows that structural coverage of the BMI1 sequence constitutes 59.5% of its total length whereas the remaining 40.5% belongs to the “dark proteome” category, typically defined by the presence of intrinsic disorder (Hu et al., 2018; Kulkarni and Uversky, 2018; Uversky, 2018).

### Pathway Proposed Downstream of BMI1 Silencing in H441 Cells

**FIGURE 7:**
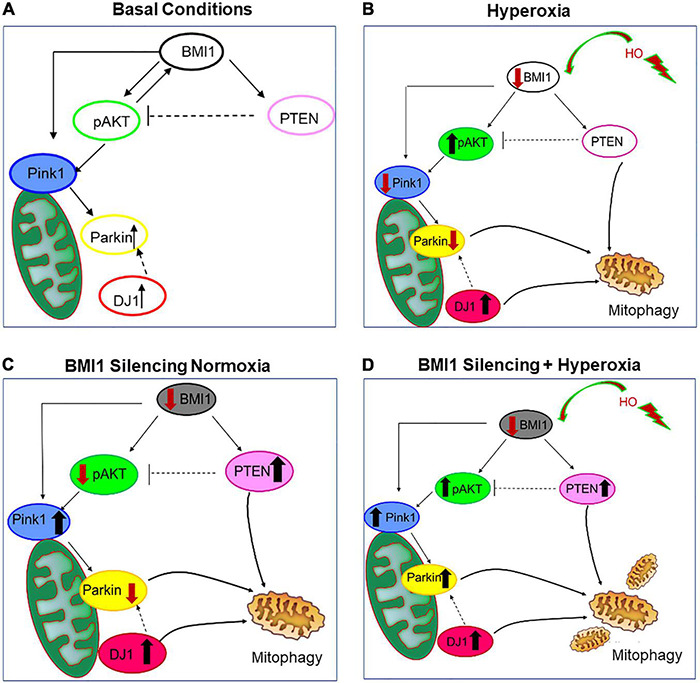
Pathway proposed downstream of BMI1 silencing in H441 cells. **(A)** Basal conditions, schematic representation of protein expression under normoxia, and normal expression of BMI1. **(B)** Hyperoxia, protein expression in H441 cells downstream of decreased expression of BMI1. **(C)** Representation of protein expression downstream of BMI1 silencing in normoxia. **(D)** The BMI1 Silencing + Hyperoxia panel represents the effects of BMI1 silencing in hyperoxia. Hyperoxia was defined as cell exposure to concentrations of oxygen higher of 80% for 48 h.

After analysis of protein expression levels, we hypothesized a pathway downstream of BMI1. Table 2 and Figure 7 summarize the different changes occurring in the regulatory proteins of mitochondrial function after BMI1 silencing in normoxic and hyperoxic conditions. While we did not elucidate the mechanism by which BMI1 silencing worsens mitochondrial damage caused by hyperoxia, we showed changes in DRP1, PTEN, Parkin, and pAKT protein levels that could be related to the observed mitochondrial dysfunction and mitophagy.

## Discussion

Hyperoxia induces mitochondrial damage, cell death, and DNA fragmentation (Kolliputi and Waxman, 2009a,b; Waxman and Kolliputi, 2009; Kolliputi et al., 2010), all characteristic signs of ALI. Based on this description, HALI represents a useful approach to study ALI/ARDS *in vivo* and *in vitro*. Diminished expression of BMI1 is associated with an increase in mitochondrial ROS production, ETC alterations, and activation of the DDR pathway (Liu et al., 2009; Bhattacharya et al., 2015; Banerjee Mustafi et al., 2016). This study provides new insight into mitochondrial dysfunction in ALI.

This research shows that mitochondrial biogenesis markers can be regulated by BMI1, and that changes in the levels of proteins involved in this dynamic process are possibly due to a compensatory mechanism to evade mitophagy and cell death (Table 2 and Figure 2). The expression of DRP1 increases under hyperoxia conditions. DRP1 is crucial to mitochondrial fission and its levels increase to remove damaged mitochondrial through mitophagy. Consequently, its degree of expression is a normal aspect of cell metabolism and mitochondrial dynamics, but also a notable compensatory response for disease and cell death (Ni et al., 2015; Xie et al., 2015; Tilokani et al., 2018; Kosmider et al., 2019). It is necessary to maintain a balance between the mitochondrial fusion and fission proteins for mitochondrial health and biogenesis. Impairment of mitochondrial fusion and fission effectively increase mitophagy in AECII, as has been observed in lung emphysema and Chronic Obstructive Pulmonary Disease (COPD) (Kosmider et al., 2019). These findings show an imbalance between the fusion proteins, OPA1, MFN1, and MFN2, and fission protein, DRP1 (Table 1 and Figure 2). It illustrates a net shift in the net activity of the mitochondrial network to favor mitophagy. Furthermore, Mfn1 and OPA1 have significant differences in mRNA expression which was observed via qRT-PC analysis of transcript levels (Figure 3C and Table 3).

Previous studies have analyzed the involvement of the PI3K-AKT pathway in ALI, either as controlling damage and activating survival or inducing apoptosis and cell death. Kolliputi and Waxman (2009b) demonstrated that IL-6 overexpression protects against HALI via the PI3K-AKT pathway. In addition, Shi et al. (2019) found that the PI3K/AKT pathway regulates mitochondria and attenuates endotoxin-induced ALI by heme oxygenase-1 (HO-1) induction, which protects the cell from the damage caused by sepsis. In the current study, no changes in the AKT transcript levels were observed in response to oxygen concentration or BMI1 silencing (Figure 3A). However, there were changes in the expression of pAKT (Ser 473) mediated by hyperoxia and BMI1 silencing (Table 1 and Figure 2). The increase in the levels of AKT phosphorylated at Ser473 is linked to cell protection (Kim et al., 2011; Murata et al., 2011). It could be a cellular response to injury caused by hyperoxia, but this response is insufficient when BMI1 silencing is also added (Figure 2B). Western blot data show that PTEN levels increased in a BMI1 silencing dependent manner (Figure 2 and Table 1). BMI1 and PTEN have two known methods of regulation, wherein BMI1 over-expression inhibits PTEN expression (at least in nasopharyngeal epithelial cells) (Song et al., 2009), and, in turn, PTEN may abrogate BMI1 function and expression in cancers, such as prostate cancer or cardiac fibroblasts (Fan et al., 2009; Kim et al., 2011; Yang et al., 2018, 2019). PTEN catalyzes the dephosphorylation of PIP3 (phosphatidylinositol-3,4,5-trisphosphate) to produce PIP2 (phosphatidylinositol4,5-biphosphate), thereby suppressing the Akt/PKB signaling pathway. This regulates cell growth and survival and is linked to mitochondrial function and metabolism (Fan et al., 2009; Gupta et al., 2017). These results corroborate the pivotal role that BMI1 plays in cell survival and renewal and highlight the importance of BMI1 normal expression in lung epithelia in response to the injury caused by hyperoxia.

The effect of BMI1 on expression of Pink1, Parkin, and DJ1 is of special interest as well. In fact, these proteins are involved to ubiquitination and mitophagy and are associated with the pathogenesis of Parkinson’s disease (Whitworth and Pallanck, 2009; Gupta et al., 2017; Salazar et al., 2018; Chia et al., 2020). The impact of alteration in the expression levels of Pink1/Parkin has been studied in LALI and demonstrate that these proteins, due to their role as homeostatic regulators of mitophagy, are necessary to respond in sepsis (Zhang et al., 2020). This study helps establish the significant impact of both BMI1 silencing and hyperoxia on the increase in the expression of Pink1 and Parkin, which, in turn, induces more mitochondrial dysfunction and mitophagy (Figure 2 and Tables 1, 2). Dey et al. (2016) demonstrated that BMI1 depletion induced mitophagy and cell death via necroptosis in an ovarian cancer cell line. The qRT-PCR results (Tables 3, 4 and Figure 3) suggest that enhanced changes in the transcript levels may be mediated by oxygen concentration rather than BMI1 silencing, and the impact of the latter is more evident at the protein level. The levels of transcripts for the NLRP3 inflammasome, BCL2, and NRF2 agree with the results of previous studies reported in HALI by our group (Waxman and Kolliputi, 2009; Kolliputi et al., 2010; Fukumoto et al., 2013).

Measurement of oxygen consumption is a method used to assess mitochondrial functionality and respiration in cells or even in isolated viable mitochondria (Xu et al., 2014). In this study, a new method to obtain live mitochondria from H441 lung epithelial cells was standardized; OCR was determined in complete H441 cells and in live mitochondria (from H441 cells) isolated after silencing and hyperoxia exposure. Analysis of OCR in both cells and isolated mitochondria revealed the same respiratory profile (Figure 4). H441 cells and mitochondria non-silenced in normoxia conditions had a high basal respiration, ATP production, and energetic phenotype (Figures 4C–F). Previously, it has been reported that hyperoxia reduces the rate of mitochondrial respiration in MLE-12 (mouse lung epithelial cells) (Das, 2013). However, in the current study, BMI1 silencing and hyperoxia were a lethal combination for mitochondrial health, leading cells to seek alternate energy resources through the glycolytic pathway or inducing senescence in cells and in isolated mitochondria (Figures 4A,B).

The tricarboxylic acid cycle (Krebs cycle) is the major source of aerobic energy and cellular metabolism (Her et al., 2015). Therefore, evaluation of the enzymatic activity of several enzymes involved in the TCA, such as fumarase, aconitase, and citrate synthase, was evaluated as a marker for oxidative stress (Nulton-Persson and Szweda, 2001; Velsor et al., 2006). A significant decrease in enzymatic activity after the silencing of BMI1 further worsened following the damage caused by hyperoxia (Figure 5). A decrease in aconitase activity with constant maintenance of fumarase activity is suggestive of oxidative stress (Velsor et al., 2006). Mitochondria from silenced cells had lower aconitase activity (0.82 nmol/min/mL) after hyperoxemia compared with mitochondria from normoxia and non-silenced cells (1.33 nmol/min/mL); with fumarase activity, the values were 0.68 nmol/min/mL and 0.72 nmol/min/mL, respectively, even though the changes in fumarase were significantly different between control siRNA NO vs. BMI1 siRNA HO groups, indicating an increment in oxidative stress.

A double fluorescence staining was performed to co-localize ROS production and mitochondrial oxidative stress. BMI1 silenced H441 cells produced more ROS and had more oxidative stress than non-silenced cells (Figures 6A,B). CellROX^®^ green is a fluorogenic reagent designed to measure ROS in live cells. In the reduced state, the green emission is weak, and after oxidation by ROS and subsequent binding to DNA, the emission becomes brighter. The images indicate higher ROS levels after hyperoxia and BMI1 silencing. Mitosox^®^ was used to measure ROS production in the form of superoxide ion, which results from the ETC. The latter increases the peroxide H_2_O_2_ production (superoxide also inactivates aconitase through oxidation of its iron-sulfur core) (Mourmoura et al., 2013). This agrees with the findings of this study (Figures 6C,D), where the silencing of BMI1 induced more ROS production than in the non-silenced cells, as evidenced by the red fluorescence *p* = 0.0080. Furthermore, the inactivation of aconitase shows oxidative stress and mitochondrial dysfunction.

The remarkable multifunctionality of BMI1 can be explained by its high binding promiscuity associated with high levels of intrinsic disorder as was explained in Supplementary Appendix and Supplementary Figure 1.

We conclude that BMI1 silencing has a deleterious and additive effect that exacerbates hyperoxia damage caused in H411 epithelial lung cells by inducing more mitochondrial damage and dysfunction, not only in terms of protein activation but also in the cellular and mitochondrial capacity for energy production, metabolism (TCA cycle), production of ROS, and oxidative stress response. Therefore, a model is proposed (Figure 7) outlining a possible pathway to induce mitophagy downstream of BMI1. The analysis of the signaling pathway proposed frames two possible therapeutic targets for the ALI/ARDS treatment: 1. recover the expression of BMI1 to avoid oxidative stress and mitochondrial dysfunction in lung epithelial cells and 2. Use the mitochondria as a therapeutic target to treat the disease. This study is limited in part due to the use of H441 cells. Future studies would use primary AECII cells or human epithelial lung cells, and this would be a physiologically relevant model for BMI1 studies.

## Materials and Methods

### Cell Culture

Three different lung cell types were grown in a humidified incubator and were exposed to normoxic [O_2_ = 21%] and hyperoxic conditions [O_2_ > 90%] (in hyperoxia chamber) for different time points (6–72 h) to elucidate if the expression of BMI1 in lung cells changes under hyperoxia conditions. **1. H441 lung epithelial cells** (this cell line has features of AECII cells and Clara cells, HTB-174™; American Type Cellular Collection, Rockville, MD), grown in RPMI 1,640 medium supplemented with 10% FBS and 1% antibiotic (penicillin-streptomycin)/antimycotic (amphotericin B) solution. H441 cells between passages 7–20 were used for all the experiments. **2. Human Lung Microvascular Endothelial Cells** (HMVEC-L) cultured in Microvascular Endothelial Growth Medium BulletKit™ (EGM™-2 MV) prepared according to the manufacturer’s protocol (Lonza, Walkersville, MD; Catalog #: CC-2527). In the current study, the HMVEC-L cells were used between passages 4–10. **3. Small Airway Epithelial Cells** (SAEC) maintained in Small Airway Growth Medium BulletKit™ (SAGM™ SingleQuots™) following manufacturer’s instructions (Lonza, Walkersville, MD; Catalog #: CC-2547), these cells were used between passages 4–10. All cells were grown in humidified incubator at 37°C and 5% CO_2_.

H441 epithelial cells, HMVEC-L and SAEC primary cells at ≈50–60% of confluency were exposed to high oxygen concentration (> 80%) for 6, 12, 24, 48, and 72 h in hyperoxia (HO) chamber or 48 h to normoxia (NO) conditions (21% O_2_). Further, cells were washed with cold 1 × PBS and trypsinized (Trypsin-EDTA 0.025%) for 5 min by incubating at 37°C. Cells were then harvested completing 10 ml with cold 1 × PBS by centrifuging at 1,500 rpm for 5 min. The supernatant was discarded, and the pellet was re-suspended in 1 ml of cold PBS 1 × and centrifuged again at 1,500 rpm for 5 min. The resulting pellet was stored at -80°C until further use.

### BMI1 Silencing

H441 cells grew in 100 mm precoated petri dishes until ≈60% of confluency to silence BMI1 using small interfering ribonucleic acid (siRNA). H441 cells with the desired confluency were transfected using siRNA from Dharmacon™ for BMI1™ (sense sequence was 5′GAACAGAUUGGAUCGGAAA3′) and control (scrambled or NTC -non target control-) siRNA (sense sequence was 5′GACUUCCGUCGACAUUAUU3′). The silencing was done each time with three replicates per treatment using Lipofectamine 2,000 (Thermo Fisher Scientific, Inc.) and BMI1 siRNA or control siRNA following manufacturer’s recommendations. A total of four groups were used for all the experiments as follow: 1 Control siRNA normoxia = normoxic conditions + scramble siRNA (experiment control). 2 BMI1siRNA normoxia = normoxic conditions + BMI1 silencing (evaluates the effect of silencing BMI1 in normal conditions of oxygen). 3 Control siRNA hyperoxia = hyperoxic conditions + scramble siRNA (to evaluates the effect of injury -hyperoxia damage- in BMI1 expression). 4 BMI1siRNA hyperoxia = hyperoxic conditions + BMI1 silencing (additive effect between the injury and the genetic modification, it evaluates the effect of silencing BMI1 in hyperoxia damage). After 18 h, the medium was changed to RPMI-supplemented medium. Cells silenced for BMI1 and non-silenced were exposed to the normoxic and hyperoxic conditions for 48 h in humidified incubator supplemented with 5% CO_2_ (all the experiments considered as hyperoxia were performed by 48 h of exposure to O2 higher tha 80%. After incubation at normal and high oxygen levels, H441 cells were trypsinized, as was described in the cell culture methods for experiments with live cells (enzyme activity assays, life mitochondria isolation, OCR) and the cells processed immediately. For protein or RNA isolation, pellets were stored at -80°C until future use.

### Protein Isolation and Western Blot

**Whole cell lysate (WCL)** was isolated from cells adding 200 μl of protein isolation buffer (150 mM NaCl, 50 mM Tris, and 0.5% NP40, pH 7.4 supplemented with protease and phosphatase inhibitors 1:100) and thermic shock induced by thawing and freezing cells three times. The WCLs were sonicated for 5 min (pulses of 15/10 s) at 50% amplitude in Qsonica Q700 sonicator. Next, the lysates were centrifuged for 15 min at 4°C × 21,000 g and supernatant collected in low binding protein tubes and stored at -80C. Protein quantification was conducted using a BCA protein assay following manufacture’s recommendations (Thermo Fisher Scientific, Inc. #23225, Pierce, Rockford, Waltham, MA).

### Western Blot Analysis

Protein expression levels were analyzed by loading 10 μg of whole cell lysate (WCL) from BMI1-silenced and non-silenced cells exposed to NO and HO conditions on 10% SDS-PAGE or in gradient gels (4–20%) for separating the lower molecular weight proteins. SDS-pages of 15 wells were used to run the proteins, in each gel were put three independent replicates of each condition (C-NO, BMI1-NO, C-HO, BMI1-HO). The proteins were transferred to PVDF membrane, blocked in 5% BSA in TBST and exposed to primary antibodies and incubated overnight at 4°C. Following washes, secondary antibody was added, according to each primary antibody, followed by the developing with chemiluminescent Kwik quant ECL solution (Kindle biosciences, Greenwich, CT), ECL or Femto (Thermo Fisher Scientific, Inc. #32209 and #34095, Pierce, Rockford, Waltham, MA) (see Supplementary Material for list of antibodies). The visualized proteins were quantified by ImageJ (NIH, Bethesda, MD), and the ratio of protein to its loading control (β-actin) was calculated using the Image J software. The results were saved in excel data sheets. The silencing experiments were performed independently several times during this study. The representative, western blot images are showing in Figures 1–3. Results of proteins expression were analyzed for the four groups together as shown in Table 1; the figures showing separately normoxia and hyperoxia data to reader better understanding. Two different blots were quantified per each protein (total of six independent replicates), and data represents the means ± SEM. *n* = 3 per group in every blot.

### Live Mitochondrial Isolation and Mitochondrial Proteins

Live mitochondria were isolated from H441 cells modifying and adjusting the protocol described in Hartwig et al. (2015) for lung cells. Live mitochondria were isolated in homogenization buffer containing sucrose, EGTA, mannitol, DTT, NaCl, and KCl by ultra-centrifugation in a sucrose gradient prepared in the homogenization buffer at concentrations of 2, 1.5, and 1 M. The mitochondria were obtained from the middle layer after several cold ultra-centrifugation steps (mitochondrial fraction). Mitochondria were resuspended in the cell growth medium for the OCR experiment or in resuspension buffer for protein isolation. Mitochondrial proteins were quantified with Pierce™ Detergent Compatible Bradford Assay Reagent and then kept frozen at −80°C.

### Real-Time Quantitative Polymerase Chain Reaction

Total RNA was isolated from H441cells (silenced and non-silenced for BMI1 and exposed to NO and HO conditions) using RNeasy Mini Kit from Qiagen (Germantown, MD), purified with RNase-free DNase set (Qiagen), dissolved in RNase-free water and stored at -80°C. For reverse transcription PCR (RT-PCR), iScript cDNA synthesis Kit (BioRad, Hercules, CA) was used following the manufacturer’s instructions and 1 μg of total RNA was reverse transcribed. To determine relative expression levels of mRNA, quantitative RT-PCR was performed using cDNAs and TaqMan qRT-PCR probes: BMI1 (Hs00995536_m1), β-ACTIN (Hs01060665_g1), TIM M23 (Hs00197056_m1), MFN1 (Hs00966851_m1), MFN2 (Hs j00208382_m1), OPA1 (Hs01047013_m1), DRP1 (Hs015 52605_m1), FIS1 (Hs00211420_m1), PTEN (Hs02621230_s1), AKT (Hs00178289_m1), PINK1 (Hs00260868_m1), PAR KIN (Hs01038322_m1), DJ1 (Hs00994893_g1), NRF2 (Hs00975961_g1), BCL2 (Hs00608023_m1), and NLRP3(Hs00918082_m1). The qRT-PCR were set up in QuantStudio 3 PCR System (Life Technologies) according to the manufacturer’s instructions for TaqMan probes and TaqMan Fast Advanced Master Mix. Data was analyzed by ΔΔ*C*_*t*_ method using β-actin (internal calibrator).

### Measurement of Oxygen Consumption Rate

Oxygen consumption rates were determined using a Seahorse XFe96 Analyzer (Seahorse Biosciences, North Billerica, MA, United States). Briefly, live H441 cells treated with scrambled siRNA or silenced with BMI1 siRNA and exposed to NO and HO conditions were plated at 50,000 cells/well in 50 μl of growth media. The 96-well plate was centrifuged at 200 g × 2 min without brake or acceleration speed and incubated in humidified incubator with 5% CO_2_. After 1 h, growth medium was replaced with 180 μl of RPMI1 Seahorse-Agilent medium pH 7.4 and supplemented with glucose, sodium pyruvate and L-glutamine following fabricant’s instructions. H441 cells were incubated for 1 h in CO_2_ free incubator and the OCR and respiratory parameters were measured by the addition of ETC inhibitors [final concentration per well: oligomycin 1.0 μM, carbonyl cyanide-p-trifluoromethoxyphenylhydrazone (FCCP) 0.5 μM and Rotenone/Antimycin-A 0.5 μM]. For live mitochondria derived from H441 cells, mitochondria were isolated as was previously described, an aliquot was stained with Mito Tracker™ green for 5 min at final concentration of 5 μM, and the particles were quantified in Neubauer haemocytometer using 40 × objective and fluorescence microscope Olympus BX43 with camera Olympus DP21. Then, a total of 1,000,000 particles were plated in 30 μl of RPMI1 Seahorse-Agilent pH 7.4 medium supplemented with glucose, sodium pyruvate, and L-glutamine, following manufacturer’s instructions, in 96 well plate and centrifuged, as was explained for live cells, and incubated for 1 h in CO_2_ free incubator. Then, 150 μl of the same medium was add to each well. This protocol was repeated to determine OCR in live cells and in live mitochondria.

### Mitochondria Activity Assays

Enzymatic activity assays were conducted using the mitochondrial fraction following the manufacturer’s instructions for fumarase activity colorimetric assay kit MAK206 (Sigma-Aldrich), Aconitase activity assay kit MAK051 (Sigma-Aldrich), and citrate synthase activity kit MAK193 (Sigma-Aldrich). The assays were carried out with isolated mitochondrial protein using mitochondrial isolation kit for cultured cells (Thermo Fisher Scientific, Inc. #89874, Pierce, Rockford, Waltham, MA) following the manufacturer’s recommendations. Once the mitochondrial protein was obtained, it was quantified using BCA assay (standards prepared in Chaps buffer 2% in 1 × TBS). The amount of protein was equalized to 1 μg/μl in assay buffer for each activity. A total of 25–30 μg of mitochondrial protein were analyzed in each activity assay with replicates, aconitase and citrate synthase activity was expressed in nmol/min/μL (milliunits/μL-mU/μL) and fumarase nmol/min/mL (milliunits/mL- mU/mL).

### Oxidative Stress and Reactive Oxygen Species Detection

#### CellROX^®^ Green Reagent and MitoTracker^®^ Red FM

H441 lung epithelial cells were seeded on glass bottom 35 mm dishes (3 for each condition, Control siRNA NO, BMI1 siRNA NO, Control siRNA HO, BMI1 siRNA HO), silenced, and exposed to normoxia and hyperoxia as previously explained. H441 cells were stained with CellROX^®^ Green Reagent (Thermo Fisher Scientific, Inc. # C10444, Pierce, Rockford, Waltham, MA) to a final concentration of 5 μM in RPMI growth medium and incubated at 37°C; after 25 min of incubation, MitoTracker^®^ Red (Thermo Fisher Scientific, Inc. # M22425, Pierce, Rockford, Waltham, MA) from 5 μM stock concentration was added to the cells to a final concentration of 25 nM. The resulting reaction mixture was incubated for 5 more minutes for a total of 30 min of CellROX incubation. The cells were then washed using DPBS and maintained in the growth medium for imaging immediately on a Leica SP8 3X STED Laser Confocal Microscope using 60 × objective. Several confocal photographs were taken for each treatment, the best images were used to quantitation (*n*: 22–10) and they were analyzed using the ImageJ-win64.Ink Fiji.app, the data are represented as mean ± SEM.

#### MitoSOX™ Red Mitochondrial Superoxide Indicator

Lung epithelial cells H441 were plated on glass bottom 35 mm petri dishes. After 60% confluency, the cells were treated with siRNA and incubated under normoxic conditions for 48 h as mentioned previously (for this assay the cells under hyperoxia did not withstand the toxicity of staining), followed by incubation with MitoSOX™ Red (Thermo Fisher Scientific, Inc. # M36008, Pierce, Rockford, Waltham, MA) to a final concentration of 5 μM in RPMI growth medium with incubation at 37°C. At minute 10 of incubation, MitoTracker Green FM (Thermo Fisher Scientific, Inc. # M7514, Pierce, Rockford, Waltham, MA) from 5 μM stock concentration was added to cells to a final concentration of 25 nM and incubated for 5 more minutes to complete 15 min of MitoSOX™ incubation. Cells were washed with DPBS and kept in growth medium to image immediately on a Leica SP8 3X STED Laser Confocal Microscope using 60 × objective. The images were analyzed utilizing the ImageJ-win64.Ink Fiji.app.

### Statistical Analysis

The data were expressed in terms of mean ± SE. All the data were evaluated using IBM SPSS Statistics (Version 26.0 Armonk, NY). Data were analyzed using ANOVA followed by Tukey’s *post-hoc* test and multivariate analysis was conducted using two-way MANOVA followed by Tukey’s *post-hoc* test for normally distributed data and *p* < 0.05 considered statistically significant. Tables and graphics were constructed using Microsoft Excel (2008). Adobe Photoshop 2020 was used for ensemble figures, keeping the original images in adequate resolution.

## Data Availability Statement

The raw data supporting the conclusions of this article will be made available by the authors, without undue reservation.

## Author Contributions

NK, VU, MA, MB, LG, RS, and RL contributed to manuscript revision and proofreading. NK contributed to final approval of the manuscript. HH-C and NK designed the study and contributed to concept and experimental design. HH-C wrote the manuscript. HH-C and VU analyzed results, analyzed the data, and prepared the figure. HH-C and SS performed the experiments. VU conducted bioinformatics analysis, protein structure, and interactivity analysis. All authors provided intellectual input and critical feedback.

## Conflict of Interest

The authors declare that the research was conducted in the absence of any commercial or financial relationships that could be construed as a potential conflict of interest.

## Publisher’s Note

All claims expressed in this article are solely those of the authors and do not necessarily represent those of their affiliated organizations, or those of the publisher, the editors and the reviewers. Any product that may be evaluated in this article, or claim that may be made by its manufacturer, is not guaranteed or endorsed by the publisher.

## Supplementary Material

Supplementary Figure 1 Computational analysis of the prevalence and functionality of intrinsic disorder in BMI1 protein. **(A)** Crystal structure of the BMI-1-RING1B core domain complex required for the efficient ubiquitin transfer. Here, the N-terminal RING/U-box domain of BMI-1 (residues 5-101, blue structure) is co-crystallized with the N-terminal domain of the RING finger protein RING1B (residues 15-114, red structure) (PDB ID: 2H0D; PMID:16714294). **(B)** the fusion protein containing PHC2_30–64_ fragment fused to the N-terminus of the central BMI1 UBL domain (residues 130–231) presented in rainbow color, where N- and C-terminal regions are shown in blue and red colors, respectively (PDB ID: 2NA1; PMID:27827373). **(C)** Intrinsic disorder profile generated for human Polycomb complex protein BMI1 (UniProt ID: P35226) by DiSpi web-crawler. Outputs of different commonly used disorder predictors, PONDR^®^ VLXT, PONDR^®^ VL3, PONDR^®^ VLS2B, PONDR^®^ FIT, IUPred2 (Short) and IUPred2 (Long) are shown by red, green, blue, black, orange, and pink colors, respectively. Gray shaded area represents errors evaluated for mean disorder profile (MDP) calculated by averaging profiles of individual predictors. **(D)** Functional intrinsic disorder profile of human BMI1 generated by D^2^P^2^ platform (http://d2p2.pro/) (PMID: 23203878). **(E)** STRING-based analysis of human BMI1 using the highest confidence level of 0.9.

## Abbreviations

ALI: Acute Lung Injury
HALI: Hyperoxia-induced Acute Lung Injury
LALI: Lipopolysaccharide-induced Acute Lung Injury
ARDS: Acute Respiratory Distress Syndrome
ROS: Reactive oxygen species
BMI1: B cell-specific Moloney murine leukemia virus integration site 1
ETC: Electron transport chain
NO: normoxia
HO: hyperoxia
AECI: Type I alveolar epithelial cells
AECII: Type II alveolar epithelial cells
DDR: DNA Damage Response pathway
AKT: protein Kinase B
p-AKT: phosphorylated protein Kinase B
PTEN: phosphatase and tensin homolog
mTOR: mammalian target of rapamycin
PI3K: phosphatidyl inositol 3 kinase
PINK1: PTEN-induced kinase 1
DJ1: protein deglycase DJ1
SAECs: Small Airway Epithelial Cells
HMVEC-L: Human Lung Microvascular Endothelial Cells
OPA1: mitochondrial dynamin like GTPase
DRP1: dynamin like-1 protein
ΔΨ m: mitochondrial membrane potential
OCR: oxygen consumption rate
TCA: tricarboxylic acid cycle
IDRs: intrinsically disordered regions
COPD: Chronic Obstructive Pulmonary Disease.

## References

[B1] Aggarwal S. Mannam P. Zhang J. (2016). Differential regulation of autophagy and mitophagy in pulmonary diseases. *Am. J. Physiol. Lung Cell Mol. Physiol.* 311 L433–L452. 10.1152/ajplung.00128.2016 27402690 PMC5504426

[B2] Banerjee Mustafi S. Aznar N. Dwivedi S. K. Chakraborty P. K. Basak R. Mukherjee P. (2016). Mitochondrial BMI1 maintains bioenergetic homeostasis in cells. *Faseb J.* 30 4042–4055. 10.1096/fj.201600321R 27613804 PMC5102112

[B3] Baughman J. M. Mootha V. K. (2006). Buffering mitochondrial DNA variation. *Nat. Genet.* 38 1232–1233. 10.1038/ng1106-1232 17072298

[B4] Beasley M. B. (2010). The pathologist’s approach to acute lung injury. *Arch. Pathol. Lab. Med.* 134 719–727. 10.5858/134.5.719 20441502

[B5] Bhattacharya R. Mustafi S. B. Street M. Dey A. Dwivedi S. K. (2015). Bmi-1: at the crossroads of physiological and pathological biology. *Genes Dis.* 2 225–239. 26448339 10.1016/j.gendis.2015.04.001 PMC4593320

[B6] Blank R. Napolitano L. M. (2011). Epidemiology of ARDS and ALI. *Crit. Care Clin.* 27 439–458. 10.1016/j.ccc.2011.05.005 21742210

[B7] Chapalamadugu K. C. Panguluri S. K. Bennett E. S. Kolliputi N. Tipparaju S. M. (2015). High level of oxygen treatment causes cardiotoxicity with arrhythmias and redox modulation. *Toxicol. Appl. Pharmacol.* 282 100–107. 10.1016/j.taap.2014.10.019 25447406 PMC4426884

[B8] Chia S. J. Tan E. K. Chao Y. X. (2020). Historical perspective: models of parkinson’s disease. *Int. J. Mol. Sci.* 21:2464. 10.3390/ijms21072464 PMC7177377 32252301

[B9] Chuquimia O. D. Petursdottir D. H. Periolo N. Fernández C. (2013). Alveolar epithelial cells are critical in protection of the respiratory tract by secretion of factors able to modulate the activity of pulmonary macrophages and directly control bacterial growth. *Infect. Immun.* 81 381–389. 10.1128/IAI.00950-12 23147039 PMC3536158

[B10] Cochrane C. G. Spragg R. Revak S. D. (1983). Pathogenesis of the adult respiratory distress syndrome. Evidence of oxidant activity in bronchoalveolar lavage fluid. *J. Clin. Invest.* 71 754–761. 10.1172/jci110823 6600748 PMC436926

[B11] Cox R. Jr. Phillips O. Fukumoto J. Fukumoto I. Parthasarathy P. T. Arias S. (2015a). Enhanced resolution of hyperoxic acute lung injury as a result of aspirin triggered resolvin D1 treatment. *Am. J. Respir. Cell Mol. Biol.* 53 422–435. 10.1165/rcmb.2014-0339OC 25647402 PMC4566067

[B12] Cox R. Jr. Phillips O. Fukumoto J. Fukumoto I. Parthasarathy P. T. Mandry M. (2015b). Resolvins decrease oxidative stress mediated macrophage and epithelial cell interaction through decreased cytokine secretion. *PLoS One* 10:e0136755. 10.1371/journal.pone.0136755 26317859 PMC4552682

[B13] Crapo J. D. Barry B. E. Foscue H. A. Shelburne J. (1980). Structural and biochemical changes in rat lungs occurring during exposures to lethal and adaptive doses of oxygen. *Am. Rev. Respir. Dis.* 122 123–143. 10.1164/arrd.1980.122.1.123 7406333

[B14] Crapo J. D. Peters-Golden M. Marsh-Salin J. Shelburne J. S. (1978). Pathologic changes in the lungs of oxygen-adapted rats: a morphometric analysis. *Lab. Invest.* 39 640–653. 739764

[B15] Das K. C. (2013). Hyperoxia decreases glycolytic capacity, glycolytic reserve and oxidative phosphorylation in MLE-12 cells and inhibits complex I and II function, but not complex IV in isolated mouse lung mitochondria. *PLoS One* 8:e73358. 10.1371/journal.pone.0073358 24023862 PMC3759456

[B16] Dey A. Mustafi S. B. Saha S. Kumar Dhar Dwivedi S. Mukherjee P. Bhattacharya R. (2016). Inhibition of BMI1 induces autophagy-mediated necroptosis. *Autophagy* 12 659–670. 10.1080/15548627.2016.1147670 27050456 PMC4836029

[B17] Dong Q. Chen L. Lu Q. Sharma S. Li L. Morimoto S. (2014). Quercetin attenuates doxorubicin cardiotoxicity by modulating Bmi-1 expression. *Br. J. Pharmacol.* 171 4440–4454. 10.1111/bph.12795 24902966 PMC4209150

[B18] Dunker A. K. Brown C. J. Lawson J. D. Iakoucheva L. M. Obradovic Z. (2002a). Intrinsic disorder and protein function. *Biochemistry* 41 6573–6582. 10.1021/bi012159+ 12022860

[B19] Dunker A. K. Brown C. J. Obradovic Z. (2002b). Identification and functions of usefully disordered proteins. *Adv. Protein Chem.* 62 25–49. 10.1016/s0065-3233(02)62004-2 12418100

[B20] Dunker A. K. Cortese M. S. Romero P. Iakoucheva L. M. Uversky V. N. (2005). Flexible nets. The roles of intrinsic disorder in protein interaction networks. *FEBS J.* 272 5129–5148. 16218947 10.1111/j.1742-4658.2005.04948.x

[B21] Dunker A. K. Lawson J. D. Brown C. J. Williams R. M. Romero P. Oh J. S. (2001). Intrinsically disordered protein. *J. Mol. Graph. Model* 19 26–59. 11381529 10.1016/s1093-3263(00)00138-8

[B22] Fan C. He L. Kapoor A. Rybak A. P. De Melo J. Cutz J. C. (2009). PTEN inhibits BMI1 function independently of its phosphatase activity. *Mol. Cancer* 8:98. 10.1186/1476-4598-8-98 19903340 PMC2777864

[B23] Ferguson N. D. Fan E. Camporota L. Antonelli M. Anzueto A. Beale R. (2012). The Berlin definition of ARDS: an expanded rationale, justification, and supplementary material. *Intensive Care Med.* 38 1573–1582. 10.1007/s00134-012-2682-1 22926653

[B24] Fukumoto J. Fukumoto I. Parthasarathy P. T. Cox R. Huynh B. Ramanathan G. K. (2013). NLRP3 deletion protects from hyperoxia-induced acute lung injury. *Am. J. Physiol. Cell Physiol.* 305 C182–C189. 10.1152/ajpcell.00086.2013 23636457 PMC3725631

[B25] Garone C. Viscomi C. (2018). Towards a therapy for mitochondrial disease: an update. *Biochem. Soc. Trans.* 46 1247–1261. 10.1042/BST20180134 30301846 PMC6195631

[B26] Goodman R. B. Pugin J. Lee J. S. Matthay M. A. (2003). Cytokine-mediated inflammation in acute lung injury. *Cytokine Growth Factor Rev.* 14 523–535. 14563354 10.1016/s1359-6101(03)00059-5

[B27] Gorman G. S. Chinnery P. F. DiMauro S. Hirano M. Koga Y. McFarland R. (2016). Mitochondrial diseases. *Nat. Rev. Dis. Primers* 2:16080. 10.1038/nrdp.2016.80 27775730

[B28] Gupta A. Anjomani-Virmouni S. Koundouros N. Dimitriadi M. Choo-Wing R. Valle A. (2017). PARK2 depletion connects energy and oxidative stress to PI3K/Akt activation via PTEN S-nitrosylation. *Mol. Cell* 65 999–1013.e7. 10.1016/j.molcel.2017.02.019 28306514 PMC5426642

[B29] Han H. Tan J. Wang R. Wan H. He Y. Yan X. (2020). PINK1 phosphorylates Drp1(S616) to regulate mitophagy-independent mitochondrial dynamics. *EMBO Rep.* 21:e48686. 10.15252/embr.201948686 32484300 PMC7403662

[B30] Hartwig S. Kotzka J. Lehr S. (2015). Isolation and quality control of functional mitochondria. *Methods Mol. Biol.* 1264 9–23. 25630999 10.1007/978-1-4939-2257-4_2

[B31] Henderson W. R. Chen L. Amato M. B. P. Brochard L. J. (2017). Fifty years of research in ARDS. respiratory mechanics in acute respiratory distress syndrome. *Am. J. Respir. Crit. Care Med.* 196 822–833. 10.1164/rccm.201612-2495CI 28306327

[B32] Her Y. F. Nelson-Holte M. Maher L. J. III (2015). Oxygen concentration controls epigenetic effects in models of familial paraganglioma. *PLoS One* 10:e0127471. 10.1371/journal.pone.0127471 25985299 PMC4436181

[B33] Herrero D. Canon S. Pelacho B. Salvador-Bernaldez M. Aguilar S. Pogontke C. (2018a). Bmi1-progenitor cell ablation impairs the angiogenic response to myocardial infarction. *Arterioscler. Thromb. Vasc. Biol.* 38 2160–2173. 29930004 10.1161/ATVBAHA.118.310778 PMC6202133

[B34] Herrero D. Tome M. Canon S. Cruz F. M. Carmona R. M. Fuster E. (2018b). Redox-dependent BMI1 activity drives in vivo adult cardiac progenitor cell differentiation. *Cell Death Dif.* 25 807–820. 10.1038/s41418-017-0022-2 PMC5864208 29323265

[B35] Hirano M. Emmanuele V. Quinzii C. M. (2018). Emerging therapies for mitochondrial diseases. *Essays Biochem.* 62 467–481. 10.1042/EBC20170114 29980632 PMC6104515

[B36] Hu G. Wang K. Song J. Uversky V. N. Kurgan L. (2018). Taxonomic landscape of the dark proteomes: whole-proteome scale interplay between structural darkness, intrinsic disorder, and crystallization propensity. *Proteomics* 18:e1800243. 10.1002/pmic.201800243 30198635

[B37] Imai Y. Kuba K. Neely G. G. Yaghubian-Malhami R. Perkmann T. van Loo G. (2008). Identification of oxidative stress and Toll-like receptor 4 signaling as a key pathway of acute lung injury. *Cell* 133 235–249. 10.1016/j.cell.2008.02.043 18423196 PMC7112336

[B38] Islam M. N. Das S. R. Emin M. T. Wei M. Sun L. Westphalen K. (2012). Mitochondrial transfer from bone-marrow-derived stromal cells to pulmonary alveoli protects against acute lung injury. *Nat. Med.* 18 759–765. 10.1038/nm.2736 22504485 PMC3727429

[B39] Johnson E. R. Matthay M. A. (2010). Acute lung injury: epidemiology, pathogenesis, and treatment. *J. Aerosol. Med. Pulm Drug Deliv.* 23 243–252. 20073554 10.1089/jamp.2009.0775 PMC3133560

[B40] Kallet R. H. Matthay M. A. (2013). Hyperoxic acute lung injury. *Respir. Care* 58 123–141. 10.4187/respcare.01963 23271823 PMC3915523

[B41] Kim J. Hwangbo J. Wong P. K. (2011). p38 MAPK-Mediated Bmi-1 down-regulation and defective proliferation in ATM-deficient neural stem cells can be restored by Akt activation. *PLoS One* 6:e16615. 10.1371/journal.pone.0016615 21305053 PMC3030607

[B42] Kolliputi N. Waxman A. B. (2009a). IL-6 cytoprotection in hyperoxic acute lung injury occurs via suppressor of cytokine signaling-1-induced apoptosis signal-regulating kinase-1 degradation. *Am. J. Respir. Cell Mol. Biol.* 40 314–324. 10.1165/rcmb.2007-0287OC 18776134 PMC2645529

[B43] Kolliputi N. Waxman A. B. (2009b). IL-6 cytoprotection in hyperoxic acute lung injury occurs via PI3K/Akt-mediated Bax phosphorylation. *Am. J. Physiol. Lung Cell Mol. Physiol.* 297 L6–L16. 10.1152/ajplung.90381.2008 19376889 PMC2711806

[B44] Kolliputi N. Galam L. Parthasarathy P. T. Tipparaju S. M. Lockey R. F. (2012). NALP-3 inflammasome silencing attenuates ceramide-induced transepithelial permeability. *J. Cell Physiol.* 227 3310–3316. 10.1002/jcp.24026 22169929 PMC3323724

[B45] Kolliputi N. Shaik R. S. Waxman A. B. (2010). The inflammasome mediates hyperoxia-induced alveolar cell permeability. *J. Immunol.* 184 5819–5826. 10.4049/jimmunol.0902766 20375306 PMC3780794

[B46] Kosmider B. Lin C. R. Karim L. Tomar D. Vlasenko L. Marchetti N. (2019). Mitochondrial dysfunction in human primary alveolar type II cells in emphysema. *EBioMedicine* 46 305–316. 10.1016/j.ebiom.2019.07.063 31383554 PMC6711885

[B47] Kulkarni P. Uversky V. N. (2018). Intrinsically disordered proteins: the dark horse of the dark proteome. *Proteomics* 18:e1800061. 10.1002/pmic.201800061 30218496

[B48] Lee J. Y. Yu K. R. Kim H. S. Kang I. Kim J. J. Lee B. C. (2016). BMI1 inhibits senescence and enhances the immunomodulatory properties of human mesenchymal stem cells via the direct suppression of MKP-1/DUSP1. *Aging (Albany NY)* 8 1670–1689. 10.18632/aging.101000 27454161 PMC5032689

[B49] Liu J. Cao L. Chen J. Song S. Lee H. I. Quijano C. (2009). Bmi1 regulates mitochondrial function and the DNA damage response pathway. *Nature* 459 387–392. 10.1038/nature08040 19404261 PMC4721521

[B50] Marti J. Hall P. Hamilton P. Lamb S. McCabe C. Lall R. (2016). One-year resource utilisation, costs and quality of life in patients with acute respiratory distress syndrome (ARDS): secondary analysis of a randomised controlled trial. *J. Intensive Care* 4:56. 10.1186/s40560-016-0178-8 27525106 PMC4982209

[B51] Massaro G. D. Gail D. B. Massaro D. (1975). Lung oxygen consumption and mitochondria of alveolar epithelial and endothelial cells. *J. Appl. Physiol.* 38 588–592. 10.1152/jappl.1975.38.4.588 1141087

[B52] McCully J. D. Cowan D. B. Emani S. M. Del Nido P. J. (2017). Mitochondrial transplantation: from animal models to clinical use in humans. *Mitochondrion* 34 127–134. 10.1016/j.mito.2017.03.004 28342934

[B53] McCully J. D. Levitsky S. Del Nido P. J. Cowan D. B. (2016). Mitochondrial transplantation for therapeutic use. *Clin. Transl. Med.* 5:16. 10.1186/s40169-016-0095-4 PMC4851669 27130633

[B54] Mourmoura E. Vial G. Laillet B. Rigaudière J. P. Hininger-Favier I. Dubouchaud H. (2013). Preserved endothelium-dependent dilatation of the coronary microvasculature at the early phase of diabetes mellitus despite the increased oxidative stress and depressed cardiac mechanical function ex vivo. *Cardiovasc. Diabetol.* 12:49. 10.1186/1475-2840-12-49 23530768 PMC3620680

[B55] Murata H. Sakaguchi M. Jin Y. Sakaguchi Y. Futami J. Yamada H. (2011). A new cytosolic pathway from a Parkinson disease-associated kinase, BRPK/PINK1: activation of AKT via mTORC2. *J. Biol. Chem.* 286 7182–7189. 10.1074/jbc.M110.179390 21177249 PMC3044975

[B56] Ni H. M. Williams J. A. Ding W. X. (2015). Mitochondrial dynamics and mitochondrial quality control. *Redox Biol.* 4 6–13. 10.1016/j.redox.2014.11.006 25479550 PMC4309858

[B57] Nulton-Persson A. C. Szweda L. I. (2001). Modulation of mitochondrial function by hydrogen peroxide. *J. Biol. Chem.* 276 23357–23361. 10.1074/jbc.M100320200 11283020

[B58] Ochiai R. (2015). Mechanical ventilation of acute respiratory distress syndrome. *J. Intensive Care* 3:25. 10.1186/s40560-015-0091-6 PMC4456061 26045965

[B59] Park I. K. Qian D. Kiel M. Becker M. W. Pihalja M. Weissman I. L. (2003). Bmi-1 is required for maintenance of adult self-renewing haematopoietic stem cells. *Nature* 423 302–305. 10.1038/nature01587 12714971

[B60] Prockop D. J. (2012). Mitochondria to the rescue. *Nat. Med.* 18 653–654. 22561816 10.1038/nm.2769

[B61] Ranieri V. M. Rubenfeld G. D. Thompson B. T. Ferguson N. D. Caldwell E. Fan E. (2012). Acute respiratory distress syndrome: the berlin definition. *JAMA* 307 2526–2533. 10.1001/jama.2012.5669 22797452

[B62] Rezoagli E. Fumagalli R. Bellani G. (2017). Definition and epidemiology of acute respiratory distress syndrome. *Ann. Transl. Med.* 5:282. 10.21037/atm.2017.06.62 PMC5537110 28828357

[B63] Ruhl A. P. Lord R. K. Panek J. A. Colantuoni E. Sepulveda K. A. Chong A. (2015). Health care resource use and costs of two-year survivors of acute lung injury. An observational cohort study. *Ann. Am. Thorac. Soc.* 12 392–401. 25594116 10.1513/AnnalsATS.201409-422OC PMC4418317

[B64] Ryter S. W. Choi A. M. (2010). Autophagy in the lung. *Proc. Am. Thorac. Soc.* 7 13–21. 20160144 10.1513/pats.200909-101JS PMC3137145

[B65] Salazar C. Ruiz-Hincapie P. Ruiz L. M. (2018). The interplay among PINK1/PARKIN/Dj-1 network during mitochondrial quality control in cancer biology: protein interaction analysis. *Cells* 7:154. 10.3390/cells7100154 30274236 PMC6210981

[B66] Shi J. Yu J. Zhang Y. Wu L. Dong S. Wu L. (2019). PI3K/Akt pathway-mediated HO-1 induction regulates mitochondrial quality control and attenuates endotoxin-induced acute lung injury. *Lab. Invest.* 99 1795–1809. 10.1038/s41374-019-0286-x 31570770

[B67] Sidramagowda Patil S. Hernández-Cuervo H. Fukumoto J. Krishnamurthy S. Lin M. Alleyn M. (2021). Alda-1 attenuates hyperoxia-induced acute lung injury in mice. *Front. Pharmacol.* 11:597942. 10.3389/fphar.2020.597942 33597876 PMC7883597

[B68] Sipmann F. S. Santos A. Tusman G. (2018). Heart-lung interactions in acute respiratory distress syndrome: pathophysiology, detection and management strategies. *Ann. Transl. Med.* 6:27. 10.21037/atm.2017.12.07 29430444 PMC5799144

[B69] Song L. B. Li J. Liao W. T. Feng Y. Yu C. P. Hu L. J. (2009). The polycomb group protein Bmi-1 represses the tumor suppressor PTEN and induces epithelial-mesenchymal transition in human nasopharyngeal epithelial cells. *J. Clin. Invest.* 119 3626–3636. 10.1172/JCI39374 19884659 PMC2786794

[B70] Tilokani L. Nagashima S. Paupe V. Prudent J. (2018). Mitochondrial dynamics: overview of molecular mechanisms. *Essays Biochem.* 62 341–360. 10.1042/EBC20170104 30030364 PMC6056715

[B71] Umbrello M. Formenti P. Bolgiaghi L. Chiumello D. (2016). Current concepts of ARDS: a narrative review. *Int. J. Mol. Sci.* 18:64. 10.3390/ijms18010064 28036088 PMC5297699

[B72] Uversky V. N. (2018). Bringing darkness to light: intrinsic disorder as a means to dig into the dark proteome. *Proteomics* 18:e1800352. 10.1002/pmic.201800352 30334344

[B73] Valiente-Alandi I. Albo-Castellanos C. Herrero D. Arza E. Garcia-Gomez M. Segovia J. C. (2015). Cardiac Bmi1(+) cells contribute to myocardial renewal in the murine adult heart. *Stem Cell Res. Ther.* 6:205. 10.1186/s13287-015-0196-9 26503423 PMC4620653

[B74] Valiente-Alandi I. Albo-Castellanos C. Herrero D. Sanchez I. Bernad A. (2016). Bmi1 (+) cardiac progenitor cells contribute to myocardial repair following acute injury. *Stem Cell Res. Ther.* 7:100. 10.1186/s13287-016-0355-7 27472922 PMC4967328

[B75] van der Lugt N. M. Domen J. Linders K. van Roon M. Robanus-Maandag E. te Riele H. (1994). Posterior transformation, neurological abnormalities, and severe hematopoietic defects in mice with a targeted deletion of the bmi-1 proto-oncogene. *Genes Dev.* 8 757–769. 10.1101/gad.8.7.757 7926765

[B76] Velsor L. W. Kariya C. Kachadourian R. Day B. J. (2006). Mitochondrial oxidative stress in the lungs of cystic fibrosis transmembrane conductance regulator protein mutant mice. *Am. J. Respir. Cell Mol. Biol.* 35 579–586. 10.1165/rcmb.2005-0473OC 16763223 PMC2643276

[B77] Viscomi C. Zeviani M. (2020). Strategies for fighting mitochondrial diseases. *J. Intern. Med.* 287 665–684. 10.1111/joim.13046 32100338

[B78] Waxman A. B. Kolliputi N. (2009). IL-6 protects against hyperoxia-induced mitochondrial damage via Bcl-2-induced Bak interactions with mitofusins. *Am. J. Respir. Cell Mol. Biol.* 41 385–396. 10.1165/rcmb.2008-0302OC 19168699 PMC2746985

[B79] Weissig V. (2020). Drug development for the therapy of mitochondrial diseases. *Trends Mol. Med.* 26 40–57. 10.1016/j.molmed.2019.09.002 31727544

[B80] Weissig V. Edeas M. (2021). Recent developments in mitochondrial medicine (Part 1). *4Open* 4:2. 10.1051/fopen/2021002

[B81] Whitworth A. J. Pallanck L. J. (2009). The PINK1/Parkin pathway: a mitochondrial quality control system? *J. Bioenerg. Biomembr.* 41 499–503. 10.1007/s10863-009-9253-3 19967438

[B82] Willmes C. (2020). Mitochondria – a powerful therapeutic target. *Trends Mol. Med.* 26 1–2. 10.1016/j.molmed.2019.10.006 31735397

[B83] Wolf D. P. Mitalipov N. Mitalipov S. (2015). Mitochondrial replacement therapy in reproductive medicine. *Trends Mol. Med.* 21 68–76. 10.1016/j.molmed.2014.12.001 25573721 PMC4377089

[B84] Wright P. E. Dyson H. J. (1999). Intrinsically unstructured proteins: re-assessing the protein structure-function paradigm. *J. Mol. Biol.* 293 321–331. 10550212 10.1006/jmbi.1999.3110

[B85] Xie Q. Wu Q. Horbinski C. M. Flavahan W. A. Yang K. Zhou W. (2015). Mitochondrial control by DRP1 in brain tumor initiating cells. *Nat. Neurosci.* 18 501–510. 10.1038/nn.3960 25730670 PMC4376639

[B86] Xu W. Janocha A. J. Leahy R. A. Klatte R. Dudzinski D. Mavrakis L. A. (2014). A novel method for pulmonary research: assessment of bioenergetic function at the air-liquid interface. *Redox Biol.* 2 513–519. 10.1016/j.redox.2014.01.004 24624341 PMC3949089

[B87] Yamada Y. Ito M. Arai M. Hibino M. Tsujioka T. Harashima H. (2020). Challenges in promoting mitochondrial transplantation therapy. *Int. J. Mol. Sci.* 21:6365. 10.3390/ijms21176365 32887310 PMC7504154

[B88] Yang W. Wu Z. Yang K. Han Y. Chen Y. Zhao W. (2019). BMI1 promotes cardiac fibrosis in ischemia-induced heart failure via the PTEN-PI3K/Akt-mTOR signaling pathway. *Am. J. Physiol. Heart Circ. Physiol.* 316 H61–H69. 10.1152/ajpheart.00487.2018 30359076

[B89] Yang X. X. Ma M. Sang M. X. Zhang X. Y. Liu Z. K. Song H. (2018). BMI-1 suppression increases the radiosensitivity of oesophageal carcinoma via the PI3K/Akt signaling pathway. *Oncol. Rep.* 39 667–678. 10.3892/or.2017.6136 29207170

[B90] Zhang Z. Chen Z. Liu R. Liang Q. Peng Z. Yin S. (2020). Bcl-2 Proteins regulate mitophagy in lipopolysaccharide-induced acute lung injury via PINK1/parkin signaling pathway. *Oxid Med. Cell Longev.* 2020:6579696. 10.1155/2020/6579696 32148654 PMC7054785

