# Supplementary figures and images for: BMI1 Silencing Induces Mitochondrial Dysfunction in Lung Epithelial Cells Exposed to Hyperoxia
Source: Front Physiol. 2022 Mar 30;13:814510. doi: 10.3389/fphys.2022.814510 (PMC9005903; doi:10.3389/fphys.2022.814510)

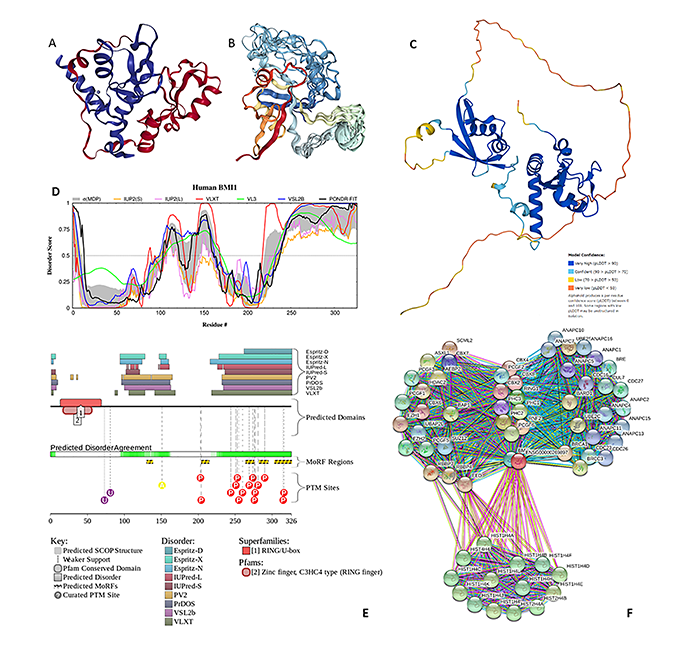

Supplement: Supplementary Figure 1 — Computational analysis of the prevalence and functionality of intrinsic disorder in BMI1 protein. (A) Crystal structure of the BMI-1-RING1B core domain complex required for the efficient ubiquitin transfer. Here, the N-terminal RING/U-box domain of BMI-1 (residues 5-101, blue structure) is co-crystallized with the N-terminal domain of the RING finger protein RING1B (residues 15-114, red structure) (PDB ID: 2H0D; PMID:16714294). (B) the fusion protein containing PHC230–64 fragment fused to the N-terminus of the central BMI1 UBL domain (residues 130–231) presented in rainbow color, where N- and C-terminal regions are shown in blue and red colors, respectively (PDB ID: 2NA1; PMID:27827373). (C) Intrinsic disorder profile generated for human Polycomb complex protein BMI1 (UniProt ID: P35226) by DiSpi web-crawler. Outputs of different commonly used disorder predictors, PONDR® VLXT, PONDR® VL3, PONDR® VLS2B, PONDR® FIT, IUPred2 (Short) and IUPred2 (Long) are shown by red, green, blue, black, orange, and pink colors, respectively. Gray shaded area represents errors evaluated for mean disorder profile (MDP) calculated by averaging profiles of individual predictors. (D) Functional intrinsic disorder profile of human BMI1 generated by D2P2 platform (http://d2p2.pro/) (PMID: 23203878). (E) STRING-based analysis of human BMI1 using the highest confidence level of 0.9. [file Image_1.TIF]
