# Supplementary material for: BMI1 Silencing Induces Mitochondrial Dysfunction in Lung Epithelial Cells Exposed to Hyperoxia
Source: Front Physiol. 2022 Mar 30;13:814510. doi: 10.3389/fphys.2022.814510 (PMC9005903; doi:10.3389/fphys.2022.814510)
Supplement: Supplementary file 2 [file Table_1.pdf]

Appendix 1. Antibodies and conditions of western blot

Antibody Name	Type	Primary Concentration	Solution	Incubation	MW (kDa)	Secondary	Secondary Concentration	Company	Reference
AKT	Rabbit	1:1000	5% BSA	ON	60	Goat anti Rb	1:5000	CST	#4691
p-AKT (Ser 473)	Rabbit	1:1000	5% BSA	ON	60	Goat anti Rb	1:5000	CST	#4060
ALDH2	Rabbit	1:100-1:500	5% Skim Milk	ON	56	Goat anti Rb	1:5000	Abcam	Ab70917
β-Actin	Rabbit	1:5000	5% BSA	45 min	45	Conjugated		CST	#4970
β-Actin	Mouse	1:5000	5% BSA	45 min	45	Conjugated		CST	#12262
BMI1	Rabbit	1:1000	5% BSA	ON	41-43	Goat anti Rb	1:5000	CST	#5856
DJ1	Rabbit	1:1000	5% BSA	ON	22	Goat anti Rb	1:5000	CST	#5933
DRP1	Rabbit	1:1000	5% BSA	ON	78-82	Goat anti Rb	1:5000	CST	#8570
Mitofusin 1	Mouse	1:1000	5% BSA	ON	84	Goat anti Ms	1:5000	Abcam	ab 57602
Mitofusin 2	Rabbit	1:1000	5% BSA	ON	80	Goat anti Rb	1:5000	CST	#9482
OPA1	Rabbit	1:1000	5% BSA	ON	86-111	Goat anti Rb	1:5000	Novusbio	NB110-55290
Parkin	Mouse	1:1000	5% BSA	ON	50	Goat anti Ms	1:5000	CST	#4211
PINK1	Rabbit	1:1000	5% BSA	ON	50-60	Goat anti Rb	1:5000	CST	#6949
PTEN	Rabbit	1:1000	5% BSA	ON	54	Goat anti Rb	1:5000	CST	#9188

ON: Over Night. CST: Cell Signaling Technologies
