# Supplementary material for: BMI1 Silencing Induces Mitochondrial Dysfunction in Lung Epithelial Cells Exposed to Hyperoxia
Source: Front Physiol. 2022 Mar 30;13:814510. doi: 10.3389/fphys.2022.814510 (PMC9005903; doi:10.3389/fphys.2022.814510)
Supplement: Supplementary file 3 [file Data_Sheet_1.pdf]

Supplemental Material

Computational analysis of intrinsic disorder predisposition of human BMI1 protein.

Methods

Intrinsic disorder predisposition of human BMI1 was analyzed using a set of commonly used per-residue disorder predictors, such as PONDR® VLXT [1], PONDR® VL3 [2], PONDR® VLS2B [3], PONDR® FIT [4], IUPred2 (Short) and IUPred2 (Long) [5; 6; 7], which were selected for their specific features. The outputs of the evaluation of the per-residue disorder propensity by these tools are represented as real numbers between 1 (ideal prediction of disorder) and 0 (ideal prediction of order). A threshold of ≥ 0.5 was used to identify disordered residues and regions in a query protein. Functional disorder profile was generated for human BMI1 by the D^2P^2 platform (http://d2p2.pro/) [8], which is a database of predicted disorder for a large library of proteins from completely sequenced genomes [8]. D^2P^2 database uses outputs of IUPred [6], PONDR® VLXT [1], PONDR® VSL2B [3], PrDOS [9], PV2 [8], and ESpritz [10]. The database is further supplemented by data concerning location of various curated posttranslational modifications and predicted disorder-based protein binding sites, known as molecular recognition features, MoRFs, which are predicted by ANCHOR algorithm [11; 12]. The interactivity of BMI1 was analyzed utilizing Search Tool for the Retrieval of Interacting Genes (STRING, http://string-db.org/) [13; 14], which generates a network of predicted associations based on predicted and experimentally validated information on the interaction partners of a protein of interest. In the corresponding network, the nodes

correspond to proteins, whereas the edges show predicted or known functional associations. Seven types of evidence are used to build the corresponding network, where they are indicated by the differently colored lines: a green line represents neighborhood evidence; a red line - the presence of fusion evidence; a purple line - experimental evidence; a blue line – co-occurrence evidence; a light blue line - database evidence; a yellow line – text mining evidence; and a black line – co-expression evidence [13; 14].

Results

We mentioned in the main manuscript BMI1 as a multifunctional protein. This multifunctionality suggests that BMI1 belongs to the group of intrinsically disordered proteins or hybrid proteins containing ordered domain(s) and functional intrinsically disordered region(s) which do not fit into the classical “one gene – one protein – one function” model. They do not have unique 3D structures but play important roles in various biological processes [15; 16; 17; 18; 19; 20; 21; 22]. It is now recognized that these proteins constitute a very substantial part of the proteomes of all living organisms [23; 24; 25; 26; 27; 28; 29; 30; 31; 32; 33; 34; 35; 36; 37; 38; 39; 40; 41] and are structurally described as highly dynamic ensembles of rapidly interconverting conformations (i.e., they remain structurally ‘floppy’ [42; 43]). This lack of fixed 3D structure, as well as their structural ‘floppiness’, define their ability to be multifunctional. Such proteins can be involved in regulation and control of various signaling processes while also serving as promiscuous binders [16; 17; 18; 43; 44; 45; 46; 47; 48; 49]. Furthermore, there is a strong link between the intrinsic disorder and pathogenesis of many diseases [50; 51], and

structure-less proteins are commonly found in cancer [52], neurodegeneration [53; 54], diabetes mellitus [55], cardiovascular disease [56], and amyloidosis [50]. To the best of our knowledge, the presence and functionality of the intrinsic disorder aspect of BMI1was not considered before this study. Bioinformatics analysis of human BMI1 protein was conducted to fill this gap.

Human Polycomb complex protein BMI1 is a component of a Polycomb Group (PcG) multiprotein PRC1-like complex required to maintain the transcriptionally repressive state of many genes that act via chromatin remodeling and modification of histones. BMI1 is a 326-residue long protein possessing a RING/U-box domain (residues 14-69) that contains a C3HC4 zinc finger motif of RING-type (residues 18-57), a Pro-Ser rich region (residues 251-326), and a nuclear localization signal (residues 81-95). The latter is a region involved in interaction with the N-terminal domain of the RING finger protein RING1B [57], and two functional regions required for interaction with polyhomeotic protein PHC2 (residues 162-182) [58] and transcription factor E4F1 (residues 164-228) [59]. Structural information is now available for the N-terminal region of BMI1 (residues 5-101) containing RING/U-box domain in complex with the N-terminal domain of the RING finger protein RING1B [57] or within the human RING1B-BMI1-UBCH5C E3-E2 complex (the PRC1 ubiquitylation module) bound to its nucleosome core particle substrate [60]. Also, an NMR solution structure was solved for the fusion protein containing $PHC2_{30–64}$ fragment fused to the N-terminus of the central BMI1 UBL domain (residues 130-231; [58]). **Figure 1S B** shows that this construct is characterized by noticeable conformational flexibility, especially in loop regions.

Intrinsic disorder predisposition of human BMI1. Data presented in the previous section suggest that structural coverage of the BMI1 sequence constitutes 59.5% of its total length whereas the remaining 40.5% belongs to the "dark proteome" category, typically defined by the presence of intrinsic disorder [61; 62; 63]. Therefore, a set of commonly utilized disorder predictors, PONDR® VLXT, PONDR® VL3, PONDR® VLS2B, PONDR® FIT, IUPred2 (Short), and IUPred2 (Long), was used to check if there was an intrinsic disorder predisposition of human BMI1. **Figure 1S C** shows the resulting disorder profile, which indicates the presence of three intrinsically disordered regions (IDRs): residues 1-5, 100-162, and 234-326. These disordered regions coincide with the regions missing structural characterization.

Potential functionality of intrinsically disordered regions in BMI1. The D^2P^2 platform (http://d2p2.pro/), which is a database of predicted disorder for a large library of proteins from completely sequenced genomes, was utilized to determine whether these IDRs have some relation to BMI1 functionality [8]. D^2P^2 uses outputs of several per-residue disorder predictors, such as IUPred, PONDR® VLXT, PrDOS, PONDR® VSL2, PV2, and ESpritz. The database is further supplemented by the data on the locations of predicted SCOP domains, conserved Pfam domains, and sites of various posttranslational modifications and predicted disorder-based protein binding sites, known as molecular recognition features, MoRFs. MoRFs are interaction-prone segments of IDRs exhibiting molecular recognition and binding functions and facilitate interactions with physiological partners. MoRFs undergo disorder-to-order transitions as result of interaction with specific partners,

and such binding-induced folding allows them to perform various biological functions [46; 64; 65; 66; 67]. However, the extended conformation, low compactness, and high solvent accessibility make IDRs excellent targets for post-translational modifications (PTMs) and proteolytic degradation, typical methods of activity regulation [16; 68; 69; 70; 71]). **Figure 1S D** represents a functional disorder profile of human BMI1 generated by D^2P^2 and shows that IDRs of this protein serve as sites of various PTMs (ubiquitination, acetylation, and phosphorylation) and also include five MoRFs (residues 130-138, 204-214, 264-275, 282-294, and 303-326). This indicates that 21% of BMI1 residues are involved in disorder-based interactions with specific partners.

Binding promiscuity of human BMI1. The multifunctionality of BMI1 is related to its ability to interact with multiple partners. **Figure 1S E** represents a dense protein-protein interaction network centered at the BMI1 generated by the online resource Search Tool for the Retrieval of Interacting Genes (STRING, https://string-db.org/). This network integrates the information on protein-protein interactions (PPIs), complements it with computational predictions, and returns a PPI network showing all possible PPIs of a query protein [13; 14]. **Figure 1S E** shows that the BMI1-centered network contains 64 nodes (proteins) connected by 1,006 edges (PPIs). In this network, the average node degree is 31.4 (i.e., on average each member of this PPI network has 41.4 interactions with other network members). The average local clustering coefficient for BMI1is 0.921. The clustering coefficient defines how close its neighbors are to being a complete clique. It is equal to 1 if every neighbor connected to a given node N_i is also connected to every other node within the neighborhood. Conversely, it is equal to 0 if no node that is connected to

a given node N_i connects to any other node that is connected to N_i. Since the expected number of interactions among proteins in a similar size set of proteins randomly selected from human proteome is equal to 137, this PPI network has significantly more interactions than expected, being characterized by a PPI enrichment p-value of $< 10^{-16}$. Therefore, this analysis shows that BMI1 can be considered a highly connected hub protein.

Discussion

The remarkable multifunctionality of BMI1 can be explained by its high binding promiscuity associated with its notable levels of intrinsic disorder. **Figure 1S** shows that although structural information is available for two domains of this protein, its actual structural coverage constitutes 59.5%, indicating that 40.5% of BMI1 is structurally uncharacterized, likely due to the high conformational flexibility of corresponding regions. In line with these observations, computational analysis revealed that almost half of human BMI1 protein (49.4%) is expected to be disordered (**Figures 1S C** and **1S D**). Furthermore, this protein contains multiple PTM sites and several disorder-based interaction sites that can undergo disorder-to-order transition at binding to specific partners (**Figure 1S D**). These data indicate that BMI1 has a strong interaction potential, and the activity of this protein is subject to regulation via PTMs. All this defines the exceptional binding promiscuity of BMI1, making it an important hub protein linking at least 63 partners (**Figure 1S E**). These observations are in line with the current knowledge on the structural principles of the organization and control of PPI networks, where the hub proteins (or their partners) are enriched in intrinsic disorder [72; 73; 74]. This defines the capability of such intrinsically disordered proteins and hybrid proteins containing ordered

domains and IDRs to serve as promiscuous interactors involved in one-to-many and many-to-one binding [75; 76].

References

[1] P. Romero, Z. Obradovic, X. Li, E.C. Garner, C.J. Brown, and A.K. Dunker, Sequence complexity of disordered protein. Proteins 42 (2001) 38-48.
[2] K. Peng, P. Radivojac, S. Vucetic, A.K. Dunker, and Z. Obradovic, Length-dependent prediction of protein intrinsic disorder. BMC bioinformatics 7 (2006) 208.
[3] K. Peng, S. Vucetic, P. Radivojac, C.J. Brown, A.K. Dunker, and Z. Obradovic, Optimizing long intrinsic disorder predictors with protein evolutionary information. J Bioinform Comput Biol 3 (2005) 35-60.
[4] B. Xue, R.L. Dunbrack, R.W. Williams, A.K. Dunker, and V.N. Uversky, PONDR-FIT: a meta-predictor of intrinsically disordered amino acids. Biochim Biophys Acta 1804 (2010) 996-1010.
[5] B. Meszaros, G. Erdos, and Z. Dosztanyi, IUPred2A: context-dependent prediction of protein disorder as a function of redox state and protein binding. Nucleic Acids Res 46 (2018) W329-W337.
[6] Z. Dosztanyi, V. Csizmok, P. Tompa, and I. Simon, IUPred: web server for the prediction of intrinsically unstructured regions of proteins based on estimated energy content. Bioinformatics 21 (2005) 3433-4.
[7] Z. Dosztanyi, V. Csizmok, P. Tompa, and I. Simon, The pairwise energy content estimated from amino acid composition discriminates between folded and intrinsically unstructured proteins. J Mol Biol 347 (2005) 827-39.
[8] M.E. Oates, P. Romero, T. Ishida, M. Ghalwash, M.J. Mizianty, B. Xue, Z. Dosztanyi, V.N. Uversky, Z. Obradovic, L. Kurgan, A.K. Dunker, and J. Gough, D(2)P(2): database of disordered protein predictions. Nucleic Acids Res 41 (2013) D508-16.
[9] T. Ishida, and K. Kinoshita, PrDOS: prediction of disordered protein regions from amino acid sequence. Nucleic Acids Res 35 (2007) W460-4.
[10] I. Walsh, A.J. Martin, T. Di Domenico, and S.C. Tosatto, ESpritz: accurate and fast prediction of protein disorder. Bioinformatics 28 (2012) 503-9.
[11] Z. Dosztanyi, B. Meszaros, and I. Simon, ANCHOR: web server for predicting protein binding regions in disordered proteins. Bioinformatics 25 (2009) 2745-6.
[12] B. Meszaros, I. Simon, and Z. Dosztanyi, Prediction of protein binding regions in disordered proteins. PLoS computational biology 5 (2009) e1000376.
[13] D. Szklarczyk, A.L. Gable, K.C. Nastou, D. Lyon, R. Kirsch, S. Pyysalo, N.T. Doncheva, M. Legeay, T. Fang, P. Bork, L.J. Jensen, and C. von Mering, The STRING database in 2021: customizable protein-protein networks, and functional characterization of user-uploaded gene/measurement sets. Nucleic Acids Res 49 (2021) D605-D612.
[14] D. Szklarczyk, A. Franceschini, M. Kuhn, M. Simonovic, A. Roth, P. Minguez, T. Doerks, M. Stark, J. Muller, P. Bork, L.J. Jensen, and C. von Mering, The

STRING database in 2011: functional interaction networks of proteins, globally integrated and scored. Nucleic Acids Res 39 (2011) D561-8.
[15] P.E. Wright, and H.J. Dyson, Intrinsically unstructured proteins: re-assessing the protein structure-function paradigm. J Mol Biol 293 (1999) 321-31.
[16] A.K. Dunker, J.D. Lawson, C.J. Brown, R.M. Williams, P. Romero, J.S. Oh, C.J. Oldfield, A.M. Campen, C.M. Ratliff, K.W. Hipps, J. Ausio, M.S. Nissen, R. Reeves, C. Kang, C.R. Kissinger, R.W. Bailey, M.D. Griswold, W. Chiu, E.C. Garner, and Z. Obradovic, Intrinsically disordered protein. J Mol Graph Model 19 (2001) 26-59.
[17] A.K. Dunker, C.J. Brown, and Z. Obradovic, Identification and functions of usefully disordered proteins. Adv Protein Chem 62 (2002) 25-49.
[18] A.K. Dunker, C.J. Brown, J.D. Lawson, L.M. Iakoucheva, and Z. Obradovic, Intrinsic disorder and protein function. Biochemistry 41 (2002) 6573-82.
[19] A.K. Dunker, and Z. Obradovic, The protein trinity--linking function and disorder. Nat Biotechnol 19 (2001) 805-6.
[20] H.J. Dyson, and P.E. Wright, Coupling of folding and binding for unstructured proteins. Curr Opin Struct Biol 12 (2002) 54-60.
[21] H.J. Dyson, and P.E. Wright, Intrinsically unstructured proteins and their functions. Nat Rev Mol Cell Biol 6 (2005) 197-208.
[22] P. Tompa, Intrinsically unstructured proteins. Trends Biochem Sci 27 (2002) 527-33.
[23] A.K. Dunker, Z. Obradovic, P. Romero, E.C. Garner, and C.J. Brown, Intrinsic protein disorder in complete genomes. Genome Inform. Ser. Workshop Genome Inform. 11 (2000) 161-71.
[24] J.J. Ward, J.S. Sodhi, L.J. McGuffin, B.F. Buxton, and D.T. Jones, Prediction and functional analysis of native disorder in proteins from the three kingdoms of life. J. Mol. Biol. 337 (2004) 635-45.
[25] N. Tokuriki, C.J. Oldfield, V.N. Uversky, I.N. Berezovsky, and D.S. Tawfik, Do viral proteins possess unique biophysical features? Trends Biochem. Sci. 34 (2009) 53-9.
[26] B. Xue, R.W. Williams, C.J. Oldfield, A.K. Dunker, and V.N. Uversky, Archaic chaos: intrinsically disordered proteins in Archaea. BMC Syst. Biol. 4 Suppl 1 (2010) S1.
[27] V.N. Uversky, The mysterious unfoldome: structureless, underappreciated, yet vital part of any given proteome. J. Biomed. Biotechnol. 2010 (2010) 568068.
[28] B. Xue, A.K. Dunker, and V.N. Uversky, Orderly order in protein intrinsic disorder distribution: disorder in 3500 proteomes from viruses and the three domains of life. J. Biomol. Struct. Dyn. 30 (2012) 137-49.
[29] P. Tompa, Z. Dosztanyi, and I. Simon, Prevalent structural disorder in E. coli and S. cerevisiae proteomes. Journal of proteome research 5 (2006) 1996-2000.
[30] M.D. Krasowski, E.J. Reschly, and S. Ekins, Intrinsic disorder in nuclear hormone receptors. Journal of proteome research 7 (2008) 4359-72.
[31] K. Shimizu, and H. Toh, Interaction between intrinsically disordered proteins frequently occurs in a human protein-protein interaction network. J Mol Biol 392 (2009) 1253-65.

[32] M.M. Pentony, and D.T. Jones, Modularity of intrinsic disorder in the human proteome. Proteins 78 (2010) 212-21.
[33] P. Tompa, and L. Kalmar, Power law distribution defines structural disorder as a structural element directly linked with function. J Mol Biol 403 (2010) 346-50.
[34] E. Schad, P. Tompa, and H. Hegyi, The relationship between proteome size, structural disorder and organism complexity. Genome biology 12 (2011) R120.
[35] H.J. Dyson, Expanding the proteome: disordered and alternatively folded proteins. Quarterly reviews of biophysics 44 (2011) 467-518.
[36] R. Pancsa, and P. Tompa, Structural disorder in eukaryotes. PLoS One 7 (2012) e34687.
[37] U. Midic, and Z. Obradovic, Intrinsic disorder in putative protein sequences. Proteome science 10 Suppl 1 (2012) S19.
[38] H. Hegyi, and P. Tompa, Increased structural disorder of proteins encoded on human sex chromosomes. Molecular bioSystems 8 (2012) 229-36.
[39] I. Korneta, and J.M. Bujnicki, Intrinsic disorder in the human spliceosomal proteome. PLoS computational biology 8 (2012) e1002641.
[40] B. Kahali, and T.C. Ghosh, Disorderness in Escherichia coli proteome: perception of folding fidelity and protein-protein interactions. Journal of biomolecular structure & dynamics 31 (2013) 472-6.
[41] T. Di Domenico, I. Walsh, and S.C. Tosatto, Analysis and consensus of currently available intrinsic protein disorder annotation sources in the MobiDB database. BMC bioinformatics 14 Suppl 7 (2013) S3.
[42] V.N. Uversky, Unusual biophysics of intrinsically disordered proteins. Biochim Biophys Acta 1834 (2013) 932-51.
[43] V.N. Uversky, The most important thing is the tail: multitudinous functionalities of intrinsically disordered protein termini. FEBS Lett 587 (2013) 1891-901.
[44] J. Habchi, P. Tompa, S. Longhi, and V.N. Uversky, Introducing protein intrinsic disorder. Chem Rev 114 (2014) 6561-88.
[45] V.N. Uversky, J.R. Gillespie, and A.L. Fink, Why are "natively unfolded" proteins unstructured under physiologic conditions? Proteins 41 (2000) 415-27.
[46] R. van der Lee, M. Buljan, B. Lang, R.J. Weatheritt, G.W. Daughdrill, A.K. Dunker, M. Fuxreiter, J. Gough, J. Gsponer, D.T. Jones, P.M. Kim, R.W. Kriwacki, C.J. Oldfield, R.V. Pappu, P. Tompa, V.N. Uversky, P.E. Wright, and M.M. Babu, Classification of intrinsically disordered regions and proteins. Chem Rev 114 (2014) 6589-631.
[47] V.N. Uversky, Natively unfolded proteins: a point where biology waits for physics. Protein Sci 11 (2002) 739-56.
[48] V.N. Uversky, What does it mean to be natively unfolded? Eur J Biochem 269 (2002) 2-12.
[49] V.N. Uversky, Protein folding revisited. A polypeptide chain at the folding-misfolding-nonfolding cross-roads: which way to go? Cell Mol Life Sci 60 (2003) 1852-71.
[50] V.N. Uversky, C.J. Oldfield, and A.K. Dunker, Intrinsically disordered proteins in human diseases: introducing the D2 concept. Annu Rev Biophys 37 (2008) 215-46.

[51] V.N. Uversky, V. Dave, L.M. Iakoucheva, P. Malaney, S.J. Metallo, R.R. Pathak, and A.C. Joerger, Pathological unfoldomics of uncontrolled chaos: intrinsically disordered proteins and human diseases. Chem Rev 114 (2014) 6844-79.
[52] L.M. Iakoucheva, C.J. Brown, J.D. Lawson, Z. Obradovic, and A.K. Dunker, Intrinsic disorder in cell-signaling and cancer-associated proteins. J Mol Biol 323 (2002) 573-84.
[53] V.N. Uversky, The triple power of D(3): protein intrinsic disorder in degenerative diseases. Front Biosci (Landmark Ed) 19 (2014) 181-258.
[54] V.N. Uversky, Intrinsic disorder in proteins associated with neurodegenerative diseases. Front Biosci (Landmark Ed) 14 (2009) 5188-238.
[55] Z. Du, and V.N. Uversky, A Comprehensive Survey of the Roles of Highly Disordered Proteins in Type 2 Diabetes. International journal of molecular sciences 18 (2017).
[56] Y. Cheng, T. LeGall, C.J. Oldfield, A.K. Dunker, and V.N. Uversky, Abundance of intrinsic disorder in protein associated with cardiovascular disease. Biochemistry 45 (2006) 10448-60.
[57] Z. Li, R. Cao, M. Wang, M.P. Myers, Y. Zhang, and R.M. Xu, Structure of a Bmi-1-Ring1B polycomb group ubiquitin ligase complex. J Biol Chem 281 (2006) 20643-9.
[58] F. Gray, H.J. Cho, S. Shukla, S. He, A. Harris, B. Boytsov, L. Jaremko, M. Jaremko, B. Demeler, E.R. Lawlor, J. Grembecka, and T. Cierpicki, BMI1 regulates PRC1 architecture and activity through homo- and hetero-oligomerization. Nat Commun 7 (2016) 13343.
[59] J. Chagraoui, S.L. Niessen, J. Lessard, S. Girard, P. Coulombe, M. Sauvageau, S. Meloche, and G. Sauvageau, E4F1: a novel candidate factor for mediating BMI1 function in primitive hematopoietic cells. Genes & development 20 (2006) 2110-20.
[60] R.K. McGinty, R.C. Henrici, and S. Tan, Crystal structure of the PRC1 ubiquitylation module bound to the nucleosome. Nature 514 (2014) 591-6.
[61] V.N. Uversky, Bringing Darkness to Light: Intrinsic Disorder as a Means to Dig into the Dark Proteome. Proteomics 18 (2018) e1800352.
[62] P. Kulkarni, and V.N. Uversky, Intrinsically Disordered Proteins: The Dark Horse of the Dark Proteome. Proteomics 18 (2018) e1800061.
[63] G. Hu, K. Wang, J. Song, V.N. Uversky, and L. Kurgan, Taxonomic Landscape of the Dark Proteomes: Whole-Proteome Scale Interplay Between Structural Darkness, Intrinsic Disorder, and Crystallization Propensity. Proteomics 18 (2018) e1800243.
[64] Y. Cheng, C.J. Oldfield, J. Meng, P. Romero, V.N. Uversky, and A.K. Dunker, Mining alpha-helix-forming molecular recognition features with cross species sequence alignments. Biochemistry 46 (2007) 13468-77.
[65] V. Vacic, C.J. Oldfield, A. Mohan, P. Radivojac, M.S. Cortese, V.N. Uversky, and A.K. Dunker, Characterization of molecular recognition features, MoRFs, and their binding partners. Journal of proteome research 6 (2007) 2351-66.
[66] A. Mohan, C.J. Oldfield, P. Radivojac, V. Vacic, M.S. Cortese, A.K. Dunker, and V.N. Uversky, Analysis of molecular recognition features (MoRFs). J Mol Biol 362 (2006) 1043-59.

[67] C.J. Oldfield, Y. Cheng, M.S. Cortese, P. Romero, V.N. Uversky, and A.K. Dunker, Coupled folding and binding with alpha-helix-forming molecular recognition elements. Biochemistry 44 (2005) 12454-70.
[68] A.L. Darling, and V.N. Uversky, Intrinsic Disorder and Posttranslational Modifications: The Darker Side of the Biological Dark Matter. Front Genet 9 (2018) 158.
[69] V. Pejaver, W.L. Hsu, F. Xin, A.K. Dunker, V.N. Uversky, and P. Radivojac, The structural and functional signatures of proteins that undergo multiple events of post-translational modification. Protein Sci 23 (2014) 1077-93.
[70] V.N. Uversky, and A.K. Dunker, Understanding protein non-folding. Biochim Biophys Acta 1804 (2010) 1231-64.
[71] L.M. Iakoucheva, P. Radivojac, C.J. Brown, T.R. O'Connor, J.G. Sikes, Z. Obradovic, and A.K. Dunker, The importance of intrinsic disorder for protein phosphorylation. Nucleic Acids Res 32 (2004) 1037-49.
[72] G. Hu, Z. Wu, V.N. Uversky, and L. Kurgan, Functional Analysis of Human Hub Proteins and Their Interactors Involved in the Intrinsic Disorder-Enriched Interactions. International journal of molecular sciences 18 (2017).
[73] Z. Dosztanyi, J. Chen, A.K. Dunker, I. Simon, and P. Tompa, Disorder and sequence repeats in hub proteins and their implications for network evolution. Journal of proteome research 5 (2006) 2985-95.
[74] A.K. Dunker, M.S. Cortese, P. Romero, L.M. Iakoucheva, and V.N. Uversky, Flexible nets. The roles of intrinsic disorder in protein interaction networks. FEBS J 272 (2005) 5129-48.
[75] V.N. Uversky, Intrinsic disorder-based protein interactions and their modulators. Curr Pharm Des 19 (2013) 4191-213.
[76] C.J. Oldfield, J. Meng, J.Y. Yang, M.Q. Yang, V.N. Uversky, and A.K. Dunker, Flexible nets: disorder and induced fit in the associations of p53 and 14-3-3 with their partners. BMC Genomics 9 Suppl 1 (2008) S1.
